# Epitaxial III–V/Si Vertical Heterostructures with Hybrid 2D‐Semimetal/Semiconductor Ambipolar and Photoactive Properties

**DOI:** 10.1002/advs.202101661

**Published:** 2021-11-11

**Authors:** Lipin Chen, Yoan Léger, Gabriel Loget, Mekan Piriyev, Imen Jadli, Sylvain Tricot, Tony Rohel, Rozenn Bernard, Alexandre Beck, Julie Le Pouliquen, Pascal Turban, Philippe Schieffer, Christophe Levallois, Bruno Fabre, Laurent Pedesseau, Jacky Even, Nicolas Bertru, Charles Cornet

**Affiliations:** ^1^ Univ Rennes INSA Rennes CNRS Institut FOTON–UMR 6082 Rennes F‐35000 France; ^2^ Univ Rennes CNRS ISCR (Institut des Sciences Chimiques de Rennes)–UMR6226 Rennes F‐35000 France; ^3^ Univ Rennes CNRS IPR (Institut de Physique de Rennes)–UMR 6251 Rennes F‐35000 France

**Keywords:** 2D topological semimetal, ambipolar properties, energy harvesting, hybrid heterostructures, III–V/Si, photo‐electro‐chemistry, photonics

## Abstract

Hybrid materials taking advantage of the different physical properties of materials are highly attractive for numerous applications in today's science and technology. Here, it is demonstrated that epitaxial bi‐domain III–V/Si are hybrid structures, composed of bulk photo‐active semiconductors with 2D topological semi‐metallic vertical inclusions, endowed with ambipolar properties. By combining structural, transport, and photoelectrochemical characterizations with first‐principle calculations, it is shown that the bi‐domain III–V/Si materials are able within the same layer to absorb light efficiently, separate laterally the photo‐generated carriers, transfer them to semimetal singularities, and ease extraction of both electrons and holes vertically, leading to efficient carrier collection. Besides, the original topological properties of the 2D semi‐metallic inclusions are also discussed. This comb‐like heterostructure not only merges the superior optical properties of semiconductors with good transport properties of metallic materials, but also combines the high efficiency and tunability afforded by III–V inorganic bulk materials with the flexible management of nano‐scale charge carriers usually offered by blends of organic materials. Physical properties of these novel hybrid heterostructures can be of great interest for energy harvesting, photonic, electronic or computing devices.

## Graphical abstract

Here, it is demonstrated that epitaxial bi‐domain III–V/Si are hybrid structures, composed of bulk photo‐active semiconductors with 2D topological semi metallic vertical inclusions, endowed with ambipolar properties. Operating III–V/Si photoelectrodes confirm that this hybrid material can by itself photogenerate and laterally separate carriers, which are efficiently extracted afterward from the photoelectric device.

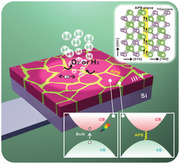

## 1 Introduction

Hybrid materials and devices have driven much research recently, addressing the most advanced issues in today's energy harvesting,^[^ ^1^ ^]^ photonic,^[^ ^2^ ^]^ sensing,^[^ ^3^ ^]^ or computing^[^ ^4^ ^]^ technologies. Among the various challenges raised, the simultaneous and ultimate control of carrier transport and optical properties in the same material remains usually tricky. On the one hand, the bandgap of semiconducting materials provides a path for electron–hole generation through light absorption, or electron‐hole recombination through light emission, especially with direct bandgap semiconductors, such as III–V, II–VI, CuInGaSe or perovskites.^[^ ^5^ ^]^ The price to pay is the necessity to adjust carefully the internal electric field and the potential profile of stacked materials needed for carrier injection or extraction, with the so‐called p‐type/n‐type architecture or using electron and hole transporting layers. Only organic devices successfully manage to combine in the same material both photo‐generation/light emission and carriers extraction or injection using bulk heterojunctions, but high yields and long lifetime are hardly reached.^[^ ^6^ ^]^ On the other hand, the absence of a bandgap in metals, semimetals or Dirac materials makes them highly interesting for transport, but they can hardly be employed as photonic or energy harvesting materials.^[^ ^7^ ^]^

Most of the classical semiconductor devices developed in the previous century were based on the combination of single usage unipolar layers, where the predominant conductive charges are holes for p‐type and electrons for n‐type, in dedicated architectures and applications. In the last past years, different research pointed out the prospects offered by ambipolar devices, where simultaneous transport of electrons and holes can be achieved, thus promoting simultaneously n‐ and p‐type characteristics. This led to recent ground‐breaking demonstrations in the field of memory or logic devices,^[^ ^8^, ^9^ ^]^ neuromorphic computing,^[^ ^10^ ^]^ sensing,^[^ ^11^ ^]^ light‐emitting devices,^[^ ^12^, ^13^ ^]^ photovoltaics,^[^ ^14^ ^]^ or photo‐electrochemical cells.^[^ ^15^ ^]^

In this work, we demonstrate experimentally and theoretically that epitaxial bi‐domain III–V/Si vertical heterostructures are hybrid structures, composed of bulk semiconductors, with 2D topological semi‐metallic inclusions, enabling simultaneous photo‐activity, charge separation, and ambipolar transport. A proof‐of‐concept is given through the design of various operating III–V/Si ambipolar photoelectrodes. The unusual topological properties of the 2D semi‐metallic inclusions are also discussed. The unique physical properties of these vertical comb‐like polyvalent heterostructures may be of great interest for energy harvesting, photonics, electronics or computing devices, combining high efficiency and predictable properties of inorganic materials and nano‐scale charge carriers transport management flexibility usually offered by organic materials.

## 2 Results and Discussion

**Figure 1:**
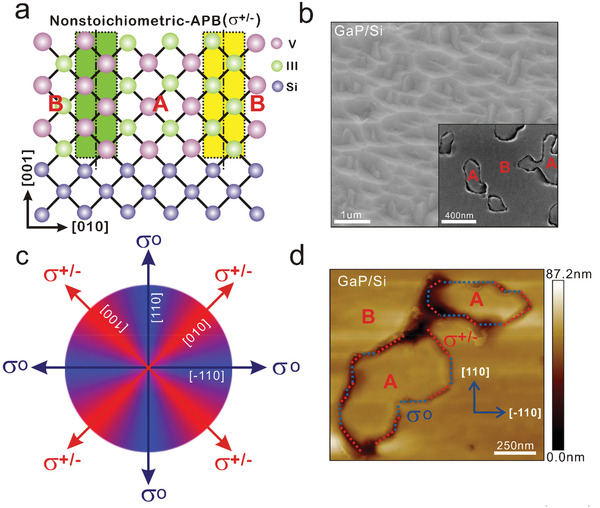
General description of antiphase boundaries in III–V/Si materials. a) Schematic of non‐stoichiometric APB atomic configuration. The APBs result from the coalescence of two domains marked by A and B (the crystal is turned by 90° in‐plane) with different phases. b) Top‐view SEM images of one bi‐domain GaP/Si sample with emerging antiphase domains. The inset corresponds to the same sample after polishing and APB developing treatment on the surface. c) Schematic diagram of in‐plane charge distribution. Red (blue) color corresponds to directions, along which vertical APBs are overall charged (neutral). d) AFM image of the bi‐domain GaP/Si sample after the APB developing treatment, revealing that APBs follow different local crystallographic directions.

Anti‐phase boundaries (APBs) are commonly formed during the epitaxy of Zinc‐Blende III–V semiconductors on a group IV substrate in the diamond phase (e.g., silicon or germanium). It originates from the two different ways that the III–V crystal may adopt to fit the group IV substrate orientation.^[^ ^16^ ^]^ As a consequence, two crystal domains with two different phases (turned by 90° in‐plane) may grow on the same substrate, separated by non‐polar III–III or V–V bonds, inside the III–V matrix, as illustrated in **Figure** **1**a; Figure S1, Supporting Information. In some crystal growth conditions, the two different domains may extend on the whole III–V material thickness, leading to a bi‐domain material configuration. Recently, large efforts were dedicated to the understanding of the formation^[^ ^16^ ^]^ and propagation^[^ ^17^ ^]^ of APBs during the epitaxial growth. With this description, early burying of the antiphase domains (APDs)^[^ ^18^, ^19^ ^]^ for lasers and photo electro chemical (PEC) devices or controlled emerging APB density for non‐linear photonics^[^ ^20^ ^]^ were achieved, showing the fine control of the crystal growth achieved. Beyond materials developments, physical properties of the specific stoichiometric APBs (with equal numbers of III–III and V–V bonds within the same APB) were recently clarified.^[^ ^21^ ^]^ It was demonstrated that the electronic bandgap is reduced by 2D electronic localization, and that, an intrinsic and strong electron‐phonon coupling arises in stoichiometric APBs and impacts the photoluminescence properties.^[^ ^21^ ^]^ Therefore, APBs should no longer be considered as usual non‐radiative recombination centers, but as a 2D homovalent singularities with specific symmetry properties in a bulk semiconducting matrix. Due to the insertion of a local translational symmetry breaking, a III–III (V–V) bond also adds two free holes (two free electrons) in the semiconductor. In the general case, the APB composition may also range from a pure vertical III–III APB to a pure V–V APB, resulting in these two extreme cases to a large excess of charges released in the lattice, as shown in Figure 1a, referred hereafter as non‐stoichiometric APBs. While different experimental works highlighted the strong influence of APBs on electrical transport properties,^[^ ^22^, ^23^, ^24^ ^]^ the intrinsic physical properties of non‐stoichiometric APBs were not explored.

In this study, bi‐domain (i.e., with emerging APBs) III–V/Si materials (GaP/Si, GaPSb/Si, GaPAs/Si) were grown on Si (001) substrate by molecular beam epitaxy (MBE). Figure 1b shows the plan‐view scanning electron microscopy (SEM) image of one as‐grown representative bi‐domain GaP/Si sample. The surface appears rough, with visible inclusions of anti‐phase materials in the main phase matrix. To further identify and study the local properties of the singularities, independently of the roughness and faceting induced by emerging APBs,^[^ ^19^ ^]^ an APB developing treatment (combining chemical mechanical polishing (CMP) and etching processes,^[^ ^20^ ^]^ as described in the Supporting Information) was used. A typical SEM image of the same sample after APB developing treatment is given in the inset of Figure 1b, showing the nice improvement of the surface smoothness in individual single‐phase domains (below 3 nm root‐mean‐square roughness), and the clear identification of emerging APBs distribution (see also Figure S2, Supporting Information, for a SEM image at a larger scale). The same procedure was applied to the different samples. For illustration, the plane‐view SEM images after APB developing treatment of one GaPSb/Si sample (Sb content ≈30%) are presented in Figure S2, Supporting Information, where the APBs emerging at the surface are shown with a denser and random honeycomb‐like lattice. For comparison, the top‐view SEM image of a n‐doped GaP wafer is shown in Figure S3, Supporting Information, from which a clean surface without APBs can be observed expectedly.

The spatial distribution of the singularities is of great importance, as it directly defines their nature. Indeed, considering a perfect vertical propagation of APBs along the [001] direction, APBs lying also along the [110] or [−110] directions will be perfectly stoichiometric, while APBs lying along [100] or [010] directions will be perfectly non‐stoichiometric (i.e., with only III–III or V–V bonds). In all the other cases, this will lead to intermediate charge states. A schematic diagram of in‐plane charge distribution is given in Figure 1c, where stoichiometric APBs lying along [110] or [−110] correspond to the neutral configuration *σ*° in blue, and non‐stoichiometric APBs lying along [100] or [010] correspond to the perfectly charged configuration *σ* ^+/−^ in red. The positive or negative nature of the charge cannot be determined here, but will be discussed in detail later in this work. Figure 1d shows the AFM image of the GaP/Si sample after APB developing treatment. While some APBs follow the [110] or [−110] directions (blue lines), and are thus expected to follow the *σ*° charge configuration, numerous other APBs are lying along other directions (red lines), and are thus expected to follow the *σ* ^+/−^ charge configuration or intermediate ones. This is in good agreement with the atomically‐resolved observations performed in previous works^[^ ^19^, ^21^ ^]^ and is not fully surprising as the APBs naturally result from the coalescence of monodomain islands.^[^ ^16^ ^]^ The simplified picture given here does not take into account the possible charge compensation effects which could occur during the growth.^[^ ^25^ ^]^ Nevertheless, it clearly evidences that bi‐domain III–V/Si samples are not composed only of *σ*°‐like APBs, but also of *σ* ^+/−^‐like ones. Note that one could certainly promote one specific APB configuration, by working in the terraces‐driven nucleation regime during III–V/Si epitaxy, with adapted and controlled Si miscut.^[^ ^17^ ^]^ The excess of charges is expected to impact significantly transport properties, which are studied in the following.

To probe the electrical conductivity and the charge carrier density, and identify the specific contribution of APBs to transport properties as well, 1 µm‐thick GaP samples were grown on different Si substrates (6°‐off or nominal ones, n‐doped or with a high resistivity (HR)) with either buried APDs (mono‐domain configuration)^[^ ^26^ ^]^ or emerging APBs (bi‐domain, such as in Figure 1b). Importantly, all these III–V layers were not intentionally doped. For some measurements, the silicon substrate was etched, and standalone GaP membranes were bonded onto a thermally oxidized silicon host wafer with an insulating adhesive polymer (benzocyclobutene polymer) layer, to better separate the transport properties of the III–V layer from the ones of Si substrate (the fabrication of bonded GaP nanomembranes is detailed in the Supporting Information). Hall effect measurements were carried out on these samples and the results are summarized in **Table** **1**. For the GaP membranes with emerging or buried APBs bonded on an insulating layer, transport physical parameters could not be determined due to their very high electrical resistance exceeding experimental capabilities. Note that a high resistance was also previously measured for a GaP layer with buried APBs on an n‐doped Si substrate.^[^ ^22^ ^]^ This result is typically expected for semiconductors with a large bandgap and non‐intentionally doped, such as GaP, when they are characterized by a standard electrical set‐up. However, charge carriers were easily injected into GaP layers with emerging APBs on Si substrates as demonstrated by Hall effect measurements. For both GaP/Si‐n and GaP/Si‐HR samples with emerging APBs, values of the measured mobility are about one order of magnitude higher than those reported in the literature for GaP/Si^[^ ^27^ ^]^ or even for bulk GaP.^[^ ^28^, ^29^ ^]^ More precisely, we observe that the measured mobility is very close to that expected for Si. This result indicates that most of the in‐plane current flows within the underlying Si substrate, without being hindered by the presence of the GaP layer. For the Si substrate, the measured sheet resistance of 170 Ω sq^−1^gives a resistivity of 5.95 Ω.cm and the sheet carrier density of 3.5 × 10^13^ cm^−2^ leads to a bulk carrier density of 1 × 10^15^ cm^−3^. These resistivity and mobility values are exactly those expected for Si with a doping concentration giving an electron density equal to the measured one.^[^ ^30^ ^]^ All these results combined together unambiguously demonstrate that carriers transport in bi‐domain III–V semiconductors occurs mostly vertically, and that the current path is related to APBs. This current path is characterized by a strong anisotropy due to a great contrast between the in‐plane APB resistivity and the out‐of‐plane APB resistivity. From our measurements, we estimate that the former is at least 10^3^ times higher than the latter.

**Table 1:** Hall effect measurements and extracted parameters (resistivity, doping concentration, and mobility) for four different samples: bi‐domain GaP membrane on insulating layer with emerging APBs; mono‐domain GaP membrane on insulating layer with buried APBs; bi‐domain GaP layer with emerging APBs on a n‐doped Si substrate; and bi‐domain GaP layer with emerging APBs on a highly resistive Si substrate. The green color represents the APBs in the schematic diagrams of the different samples. The results demonstrate that the current flows vertically through the III–V APBs and laterally through the silicon

	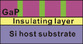	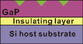	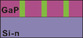	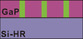	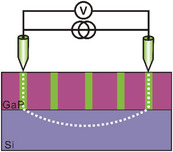
Type	Resistance >10GΩ	Resistance >10GΩ	N	N	
Sheet resistance[Ω _◻_^−1^]			170	7.8 × 10^4^	
Charge carrier sheet density [cm^−2^]			3.5 × 10^13^	8 × 10^10^	
Mobility [cm^2^ V^−1^ s^−1^]			1450	950	

**Figure 2:**
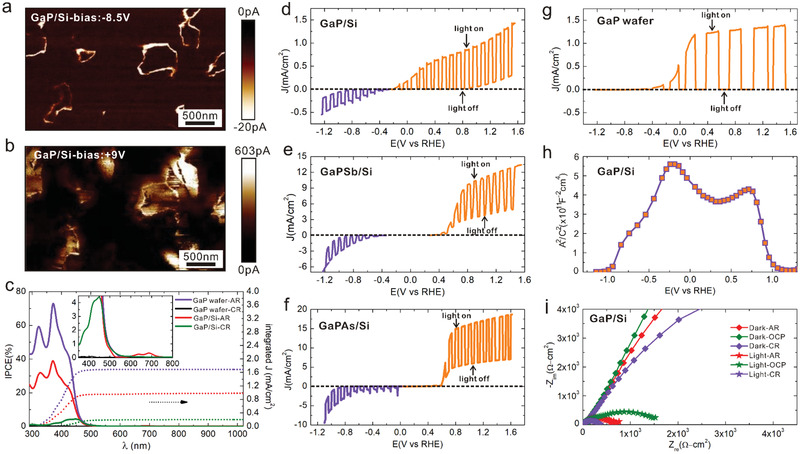
C‐AFM and PEC characterizations of III–V/Si samples with emerging APBs. a,b) C‐AFM of the bi‐domain GaP/Si:n sample under negative voltage (−8.5V) (a) and positive voltage (+9.0V) (b). c) IPCE spectra recorded at −0.74 V versus RHE (cathodic region (CR)‐green and black lines) and 1.26 V versus RHE (anodic region (AR)‐red and purple lines) for the bi‐domain GaP/Si:n (red and green lines) and GaP:n wafer (purple and black lines). The dotted lines correspond to integrated photocurrent responses and the inset are the enlarged IPCE spectra ≈350–800 nm. d–g) Chopped‐light linear sweep voltammetry curves (scan rate = 50 mV s^−1^) of bi‐domain GaP/Si:n (d), GaPSb/Si:n (e), and GaPAs/Si:n (f) samples and GaP:n wafer (g), in 1.0 m H_2_SO_4_ (pH = 0.3) electrolyte under simulated sunlight illumination (100 mW.cm^−2^, AM 1.5G). h) Mott–Schottky *C^−2^−E* plot recorded at 1 kHz for the bi‐domain GaP/Si:n sample in the dark. i) Nyquist plots (between 100 kHz and 1 Hz) of the bi‐domain GaP/Si:n sample in the dark and under illumination at different potentials: OCP, within anodic region (AR) and cathodic region (CR).

To get more local information about transport, the nano‐electronic properties of a bi‐domain GaP/n‐type Si (Si:n) sample were evaluated by conductive atomic force microscopy (C‐AFM). The surface current mapping images obtained under negative and positive biases are shown in **Figure** **2**a,b, respectively (the corresponding topography image is shown in Figure S5, Supporting Information). For both biases, a clear maximum intensity is observed where the APBs emerge, further confirming the prominent role of APBs in the charge carrier transport. This is also supported by C‐AFM measurements performed on another bi‐domain GaPSb/Si:n sample (Figure S6, Supporting Information). The observation of a current at the APB level under both positive and negative biases highlights the bidirectional conduction ability of the singularities. We also note that the maximum intensity measured for both biases cannot be quantitatively compared here due to the influence of the substrate doping.

Bi‐domain III–V/Si samples are thus composed of vertical singularities that can ensure the transport of carriers, but are also composed of monodomain bulk semiconductor grains (bounded by the singularities), whose photo‐activity should remain preserved (for absorption or emission). Therefore, the demonstration of an efficient photoelectric device with emerging APBs is conditioned by the ability to transfer the carriers from the bulk semiconducting grains to the APB singularities and vice versa. In the following, we explore the potential of bi‐domain III–V/Si materials for the fabrication of efficient photoelectrodes for water photoelectrolysis, taking advantage of the attractive characteristics offered by such materials. Indeed, solar water splitting allows converting solar energy to hydrogen through a photoelectrochemical (PEC) cell, wherein the cathodic hydrogen evolution reaction (HER) and the anodic oxygen evolution reaction (OER) occur, and provides a promising path for scalable and sustainable carbon‐free energy production and storage.^[^ ^31^, ^32^ ^]^ HER and OER occur through the transfer of photogenerated minority carriers at the solid–liquid phase, thus, for traditional bulk semiconductors, photoanodes (photocathodes) are usually associated to n‐type (p‐type) materials.

PEC characterizations were carried out in a three‐electrode cell filled with 1 m H_2_SO_4_ (measured pH = 0.3) and tested with simulated sunlight (AM 1.5G, 100 mW.cm^−2^) illumination for various bare and non‐intentionally doped bi‐domain III–V/Si materials. The chopped‐light linear sweep voltammetry (LSV) curve of the bi‐domain GaP/Si:n sample is given in Figure 2d. Interestingly, it shows both anodic and cathodic photocurrents. This unusual ambipolar behavior is of great interest for the development of fully integrated PEC cells, as it may allow the use of a single material for both photoelectrodes, which would be very convenient for the manufacturing of water splitting PEC cells. The fact that measured anodic photocurrents are higher than the cathodic ones is coherent with the C‐AFM results. For comparison, the same curve is given for a single n‐type GaP (GaP:n) wafer, which shows, as expected, only a photoanodic behavior (Figure 2g). Moreover, the voltammetry curves of bi‐domain GaPSb/Si:n and GaPAs/Si:n samples given in Figure 2e,f, reveal similar behaviors, demonstrating that ambipolar transport is not only a specific property of bidomain GaP/Si:n, but a general trend for all bidomain III–V /Si systems. Note that all these samples have been grown in similar conditions than the one used in ref. [23] for the demonstration of a GaPSb/Si photoanode, where transmission electron microscopy has been used to evidence vertical APBs. Besides, it is found that the onset potential values for cathodic photocurrent and anodic photocurrent of GaP/Si:n sample are very close and ≈ −0.3V versus reversible hydrogen electrode (RHE) (Figure 2d). This suggests an unusual distribution of carriers in bulk GaP where both electrons and holes are available almost simultaneously for the oxidation and the reduction reactions. For the GaPSb/Si:n and GaPAs/Si:n samples, the potential differences between the onsets of cathodic and anodic photocurrents are also smaller than the bandgaps of the bulk ternary compounds (Figure 2e,f). Since our surfaces are not coated with a protective layer, the photocurrents are not only representative of HER and OER but are rather the result of a kinetic competition between photocorrosion and the latter reactions.^[^ ^33^, ^34^, ^35^ ^]^ For comparison, the best performances reported in the literature with optimized III–V photoelectrodes, indicated a saturation current of ≈15 mA.cm^−2^ for a np^+^ (2 × 10^17^‐5 × 10^18^cm^−3^) junction GaAs photoanode and ≈4 mA.cm^−2^ for a n‐doped (5 × 10^17^cm^−3^) GaP photoanode by using KOH alkaline electrolyte (pH = 13.7).^[^ ^36^ ^]^ Despite the corrosion process and the far from ideal materials quality, the reached maximum photocurrent is relatively high, and close to that measured with bare III–V substrates, indicating that the photogeneration and transport are overall efficient in these devices.

For stable water photoelectrolysis, the corrosion issue is expected to be tackled effectively in a short‐term perspective by using catalytic and/or protection layers, as widely demonstrated on numerous unstable semiconductors.^[^ ^23^, ^36^, ^37^ ^]^ The large and indirect bandgap of GaP leads to relatively low light absorption and consequently to small photocurrent (as shown in Figure 2d,g). Nevertheless, with the addition of Sb or As, both the bandgap and band type of GaP‐based ternary alloys can be adjusted to increase light absorption from the solar spectrum and improve the PEC performances, as testified by the relatively high photocurrent measured for GaPSb/Si:n (30% Sb content and 1.7eV bandgap) and GaPAs/Si:n (90% As content and 1.5eV bandgap) samples. Bi‐domain GaPSb sample on p‐doped Si also shows photo‐induced ambipolar characteristic (Figure S7, Supporting Information), indicating that the intrinsic ambipolar behavior does not depend on the Si substrate doping.

Figure 2c shows the incident photon‐to‐current efficiency (IPCE) spectra of the bi‐domain GaP/Si:n sample and GaP:n wafer, recorded at 1.26V (anodic region) and −0.74V (cathodic region) versus RHE. First, the integrated photocurrents derived from the IPCE curve (0.2 mA cm^−2^ for GaP/Si:n at −0.74 V; 1.0 mA cm^−2^ for GaP/Si:n at 1.26 V; 0 mA cm^−2^ for GaP:n wafer at ‐0.74V; 1.68 mA cm^−2^ for GaP:n wafer at 1.26V) show good consistencies with those extracted from the voltammetry curves (Figure 2d,g), which validate our measurements. Second, in contrast with the GaP:n wafer, the GaP/Si:n sample shows IPCE response on both anodic and cathodic regions, which further confirms the ambipolar characteristics of the bidomain GaP/Si:n sample. Third, the IPCE of GaP/Si:n mainly increases around 550 nm, similarly to the GaP wafer, which corresponds well to the bandgap of the GaP semiconductor, indicating the carriers are mainly created by the absorption within the GaP bulk matrix. This is an important point as it already confirms that carriers are photogenerated in bulk‐like domains and then transferred to APBs, which ensure the transport. Besides, one can state that the absorption of the underlying Si substrate is not interfering in the PEC response in this case.

Additionally, electrochemical impedance spectroscopy (EIS) measurements were performed for the GaP/Si:n sample and the GaP:n wafer in contact with the same electrolyte. Figure 2h shows the Mott–Schottky (MS) plots for the GaP/Si:n sample, which exhibits both positive and negative slopes of the reciprocal of the square of capacitance versus the applied potential, revealing, once again, the simultaneous n‐ and p‐type behavior‐like of our materials.^[^ ^38^, ^39^, ^40^ ^]^ As a comparison, the MS plot measured for the GaP:n wafer is shown in Figure S8, Supporting Information, and exhibits the expected typical positive slope for an n‐doped semiconductor. From the slope, the donor density is evaluated to 2 × 10^17^ cm^−3^, which is in good agreement with the GaP wafer specifications. Finally, EIS measured with the GaP/Si sample in the dark and under illumination in the 100 kHz–1 Hz frequency range at different potentials (open circuit potential (OCP), anodic region, and cathodic region) are shown as Nyquist plots in Figure 2i. These curves suggest that the charge‐transfer resistance (*R*_ct_) (correlated with the diameters of the semi‐circles measured)^[^ ^41^ ^]^ strongly decreased under illumination and that the relatively small semi‐circles were obtained in both anodic and cathodic regimes, echoing with the ambipolar behavior. From all these PEC characterizations, we can therefore draw the following picture: bi‐domain III–V/Si materials enable (i) photogeneration of carriers in bulk monodomain semiconducting grains, (ii) carrier separation and extraction from the bulk phase to the APB singularities, (iii) ambipolar vertical transport, and (*iv*) efficient charge transfer at the solid/liquid interface.

**Figure 3:**
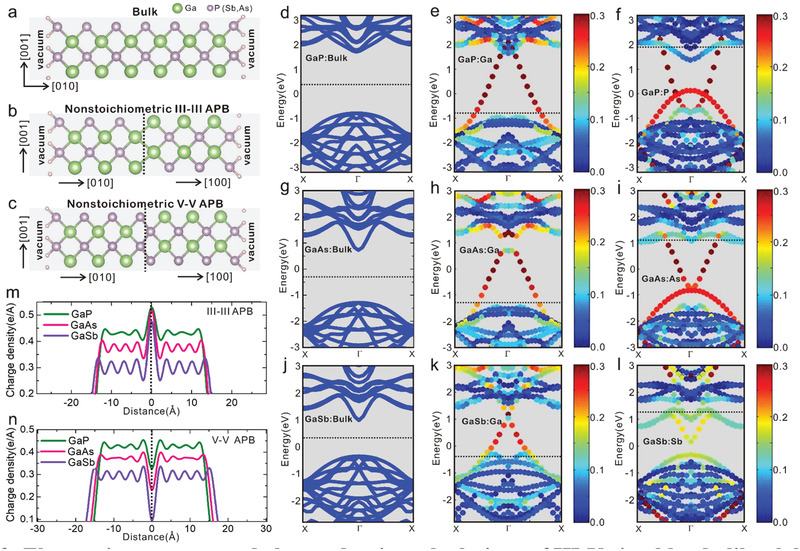
Electronic structure and charge density calculations of III–V zinc‐blende‐like slabs and non‐stoichiometric APB structures. a–c) Zinc‐blende (a), III–III APB (b), and V–V APB (c) slab structures for theoretical calculations. d–l) Calculated electronic band structures of zinc‐blende, III–III APB and V–V APB structures for GaP (d–f), GaAs (g–i), and GaSb (j–l). The APB band structures (e,f,h,i,k,l) are weighted by the spatial localization of the states at the APB atoms and the color map from blue to red underlines the increase of localization effect of the states at the APB. m,n) Charge density of III–III APB (m) and V–V APB (n) structures, integrated in the planes parallel to APBs.

For a better understanding of these original electronic and PEC properties of the APB singularities buried into III–V semiconductors, theoretical calculations were performed for III–V‐based APB structures. One of the first consequences of the intrinsic APB structure (both stoichiometric^[^ ^21^ ^]^ and non‐stoichiometric) is that, besides loss of translational symmetry, the inversion symmetry, which is absent in the bulk III–V zinc‐blende lattice, is restored locally (stoichiometric APB structure is given in Figure S9b, Supporting Information, non‐stoichiometric III–III APB in **Figure** **3**b; non‐stoichiometric V–V APB in Figure 3c). The two different kinds of APB structures designed for theoretical studies belong to different space groups: Pnnm for the stoichiometric APB structure^[^ ^21^ ^]^ and Pmma for the non‐stoichiometric APB structures. Both structures present an inversion symmetry, but the atomic motifs are different. First‐principle calculations were performed for non‐stoichiometric APB structures with GaP, GaAs, and GaSb materials. In order to investigate separately the III–III and V–V APB singularities, slab structures are constructed by adding vacuum on both sides and the surfaces were passivated with fictitious hydrogen atoms to avoid localized surface states, as shown in Figure 3b,c (called hereafter III–III and V–V APB structures). For comparison, the corresponding calculations were also done for reference III–V slab structures without APB, mimicking the bulk material (called hereafter zinc‐blende slab structures – ZBS) (Figure 3a).

Electronic band structure calculations were performed using density functional theory (DFT) along *X*(0.5,0,0)‐Γ(0,0,0)‐*X*(‐0.5,0,0) k‐path, where the axes *x*, *y*, *z* of the reciprocal space correspond to the [101], [10‐1], [010] crystallographic directions, based on the Heyd–Scuseria–Ernzerhof (HSE) hybrid functional. The band structure plots of the III–III and V–V APB structures for GaP, GaAs, and GaSb are shown in Figure 3e,f,h,i,k,l, where the color scale indicates the spatial localization of the electronic state in the APB plane. Note that the common k‐paths chosen in all the cases for the sake of comparison lay in the APB plane (Figure 3b,c) to illustrate the effects of the 2D electronic dispersion. Figure 3d,g,j shows the band structure of the reference III‐V ZBS for GaP (Figure 3d), GaAs (Figure 3g), and GaSb (Figure 3j), respectively. Compared to the normal bulk structures (unit cell with two atoms) with a zinc‐blende lattice (Figure S10, Supporting Information), the ZBS used to mimic bulk properties exhibit larger bandgaps due to quantum effects, and more bands resulting from the band folding effect. The Fermi energy levels are determined based on the Fermi energy calculations. Compared to the reference III–V ZBS, all the III–III and V–V APB structures introduce metallic states in the gap, localized on the APB atoms, which is a direct consequence of the specific 2D topology of the APB. Additionally, Figure 3m,n shows the planar averages of the charge densities integrated parallel to the APB plane. In order to show the charge distribution more clearly, longer III–III and V–V APB structures were used for charge density calculations, as shown in Figure S11, Supporting Information. The charge density increases on III–III APB and drops on V–V APB, revealing that more electrons (holes) are localized on the III–III (V–V) APB planes, and suggesting that the non‐stoichiometric APB singularities behave as charged domain walls.

**Figure 4:**
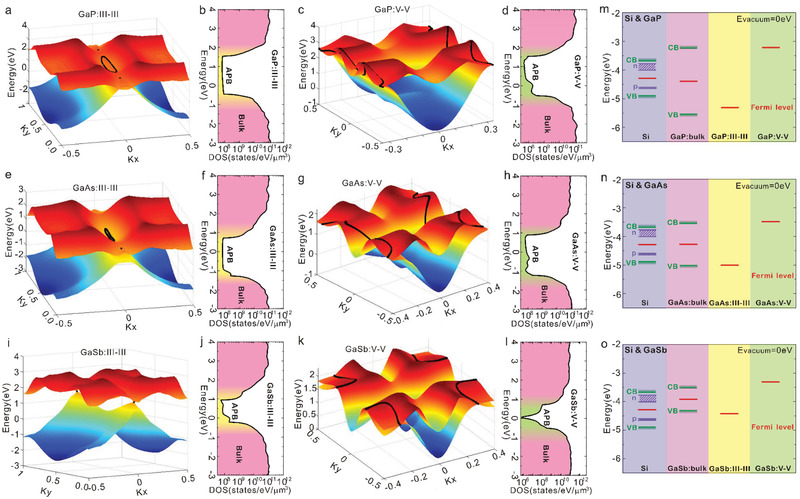
3D band structure, density of states, and Fermi energy calculations for non‐stoichiometric APB structures. a–l) 3D plots of CBM and VBM of the III–III and V–V APB slab structures, extracted from first‐principle tight‐binding model (a,c,e,g,i,k) and realistic densities of states in log scale for the III–III and V–V APB structures using actual APB densities (b,d,f,h,j,l). m–o) Fermi energy alignments of III–V zinc‐blende, III–III, and V–V APB structures with n‐doped and p‐doped Si corresponding to GaP/Si (m), GaAs/Si (n), and GaSb/Si (o) samples. All the energy levels are shifted with the vacuum energy set to 0 eV.

To further explore the intrinsic semimetal nature of these APB structures, first‐principle tight‐binding models were constructed by projecting the electronic states onto Wannier orbitals. This analysis was performed on top of state‐of‐the‐art DFT calculations at the HSE level to get a precise insight into the topological properties.^[^ ^42^ ^]^ The band structures along the X‐Γ‐X k‐path of III–III and V–V APB structures for GaP, GaAs, and GaSb were extracted, as shown in Figure S13, Supporting Information, with a very good correspondence to the initial DFT calculations (Figure 3). The density of states (DOS) of the realistic hybrid structures were then calculated, as shown in **Figure** **4**b,d,f,h,j,l, by taking into account the experimental linear densities of APBs extracted from SEM images and by considering that the computed total DOS is essentially a superposition of 3D‐like CB (conduction band) and VB (valence band) DOS with a small 2D electronic DOS in between, filling the bandgap (see the calculation details in the Supporting Information). Such DOS distributions confirm the semi metallic nature of both III–III and V–V APBs. A large ratio of at least 10^3^ between the number of states available at a given energy in the bulk region and the number of states localized in the APBs is observed, indicating that any charge transfer from the bulk semiconductor domains to the 2D APB singularities drastically changes the filling of the APBs states and thus easily shifts its Fermi level.

Moreover, 3D plot of the VBM (valence band maximum) and CBM (conduction band minimum) for III–III and V–V APB structures corresponding to k_z_ = 0 planes for GaP, GaAs ,and GaSb were extracted in Figure 4a,c,e,g,i,k. The crossing points are marked by black dots. We can observe that for the III–III APB structures, one pair of Weyl points^[^ ^43^ ^]^ and one closed nodal ring^[^ ^44^ ^]^ around the high symmetry Y point exist. A pair of Weyl points is predicted from DFT calculations for all the bi‐domain III–V materials considered here. On the one hand, the nodal ring is predicted to vary from a large one (GaP) to small one (GaAs) and even disappears for GaSb, which may be related to the large variations of their bandgaps. On the other hand, non‐closed snakelike nodal lines^[^ ^44^ ^]^ are evidenced for the V–V APB structures. The crossing points are further confirmed by DFT enlarged band structure calculations based on the metaGGA (TB‐mBJ) potential^[^ ^45^ ^]^ (which possesses similar accuracy than computationally more expensive functionals, such as HSE, or even many‐body calculations at the GW level^[^ ^46^ ^]^) combined with band symmetry analysis (Figures S16–S23, Supporting Information). The pairs of topological Weyl points of III–III APB structures are characterized by a Berry phase equals to *π*, along a close k path in the k_z_ = 0 plane (as shown in Figure S16, Supporting Information). The band crossings are protected by a combination of the time‐reversal symmetry preserved in the III–V lattice and the spatial inversion symmetry restored at the APB.^[^ ^44^, ^47^, ^48^ ^]^ Such topological features are more generally predicted to be fingerprints of the intrinsic symmetry properties of charged domain walls related to topological nodal‐line semi‐metals.^[^ ^49^ ^]^ Deeper investigations are however needed to further explore the experimental implications of such topological predictions, but non‐stoichiometric and charged III–V APBs presented here appear as leading to a first practical device realization based on these concepts.

From the present analysis and experimental study, it is already clear that epitaxial bi‐domain III–V/Si materials are hybrid comb‐like heterostructures, composed of bulk semiconductors, with 2D semi metallic vertical inclusions, enabling simultaneously photo‐activity, charge separation, and ambipolar transport. In order to have a better hint of the promising functionalities afforded by this new class of polyvalent heterostructures for photonics, computing, or energy harvesting devices, a general overview of the related energy diagrams is necessary. Fermi energy levels of reference zinc blende, III–III and V–V APB structures were carefully determined by DFT calculations using a metaGGA (TB‐mBJ) potential^[^ ^45^ ^]^ with dense k point meshes. The Fermi energy lineups after careful calibration of the vacuum energy, as well as energetic positions of the deep energy levels and bulk energy states, are shown in Figure 4m–o, together with the VBM and CBM of zinc blende III–V bulk materials and the energy levels of Si. The VBM and CBM of Si were aligned with the ones of bulk III–Vs based on ref. [50], and the fluctuations of the Fermi level for n‐doped and p‐doped Si correspond to the range of the doping concentration of Si substrates experimentally used in this study. All the energy levels are shifted by setting the vacuum energy to 0 eV. We can find that compared to the bulk Fermi levels, the V–V APB structures have quite high Fermi levels and the III–III APB structures have much lower Fermi levels, close to the CBM and VBM of the bulk, respectively. It indicates that the two different *σ* ^+/−^ APBs are expected to behave as highly n‐doped and p‐doped semiconductors, which should dominate the ambipolar PEC properties and shed light on the MS plot with positive and negative slopes.^[^ ^38^, ^39^, ^40^ ^]^

In addition, compared to the zinc‐blende bulk materials (Figure S24, Supporting Information), stoichiometric *σ* ^0^ APB structures also introduce several localized states on the top of valence bands, shifting VBM upward and reducing the bandgap (Figure S25, Supporting Information).^[^ ^21^, ^51^ ^]^ The IPCE data shows a weak signal in the 550–750 nm range for the bi‐domain GaP/Si sample (Figure 2c), which might correspond to the absorption of stoichiometric APB structures. These *σ* ^0^ APBs may also contribute to the transport through strong electron‐phonon coupling related processes,^[^ ^21^ ^]^ but it is not considered to be the main origin of the efficient ambipolar transport properties evidenced in the present work.

**Figure 5:**
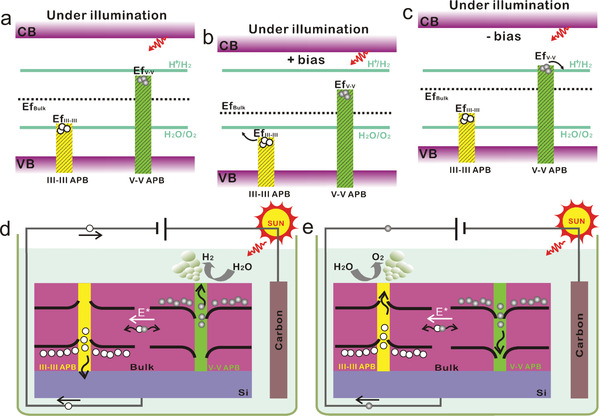
Ambipolar water splitting with bidomain III–V/Si materials. a–c) Fermi energy band diagrams of III–V bulk, III–III APB, V–V APB, and electrolyte for GaP in three cases: under illumination (a), under illumination and positive bias (b), and under illumination and negative bias (c). The yellow and green strips correspond to the occupied states of III–III and V–V APBs. d,e) Spatial schematic diagrams of the ambipolar water splitting mechanism as a photocathode (d) and as a photoanode (e) based on V–V and III–III APB singularities, respectively. The yellow and green rectangles represent the III–III and V–V APB singularities, respectively, and the black lines correspond to the CBM and VBM of the bulk III–V semiconductor.

To describe the ambipolar PEC operation of the hybrid semimetal/semiconductor bidomain III–V/Si material, from the carrier photo‐generation to the interfacial Faradaic reactions (represented in this scheme as OER and HER, although photocorrosion probably takes place), we propose the mechanism depicted in terms of band lineups for GaP (**Figure** **5**a–c), together with its spatial representation (Figure 5d,e). As described above, the Fermi levels of V–V APBs and III–III APBs are respectively close to the CBM and VBM of the bulk (Figure 4m,n,o). When the III–III and V–V APBs couple together with bulk materials, charge redistribution occurs and aligns the Fermi levels to reach equilibrium state. In particular, V–V APB structures lose electrons, resulting in the lowering of the V–V Fermi level. Meanwhile, III–III APB structures capture electrons, with an associated increase of their Fermi level. Indeed, even though the APBs introduce metallic states, the states are much less numerous than in the bulk part as shown previously with the DOS calculations (Figure 4b,d,f,h,g,l), which indicates that the Fermi level of the APB structures can easily be shifted by accepting or donating electrons without significant change of the bulk III–V Fermi level. As a consequence, the bands of the bulk III–V will bend downward at the vicinity of V–V APB and bend upward at the vicinity of III–III APB (as shown in Figure 5d,e), leading to more holes in V–V APB and more electrons in III–III APB structures. This will generate a strong in plane built‐in electric field with a direction from V–V APB pointing to III–III APB structures (white arrows in Figure 5d,e). We note that this situation is very different from that encountered with conventional inorganic semiconductors stacked structures, where the separation of photogenerated carriers is usually made vertically, with the help of the so‐called p–n architecture.

Under light illumination, the electrons and holes photogenerated in the III–V bulk matrix will then migrate to V–V and III–III APB singularities, respectively, thanks to the in‐plane built‐in electric field (as shown in Figure 5d,e). The built‐in electric field provides here the driving force for efficient photogenerated charge carriers separation. In addition, photogenerated carriers are rapidly captured by APBs due to the very short distance between neighboring APBs (<100 nm), reducing the recombination‐related losses, which shed light on the strong decrease of the charge–transfer resistance after illumination (Figure 2i).^[^ ^52^, ^53^ ^]^ With the electrons (holes) reinjected in V–V (III–III) APBs, the Fermi energy level of V–V (III–III) APBs increases (decreases) again, as shown in Figure 5a. The induced photo‐potential between these two quasi‐Fermi levels can be large due to the small DOS of metallic states. Finally, under negative bias applied vertically, the electrons in a V–V APB will be driven to the surface to generate H_2_ and holes in a III–III APB will be transfered to the Si and will further feed the counter‐electrode (Figure 5d). Under positive bias applied vertically, the holes in a III–III APB will move to the surface to generate O_2_ and the electrons in a V–V APB will be transfered to the Si and feed the counter‐electrode (Figure 5e), through conductive APB paths. This process also implies that the electrical resistance of the APBs is large enough to be able to apply a potential drop vertically along the APB. From the energy band point of view, under positive and negative bias, the Fermi energy levels of III–V APBs and bulk will shift down (Figure 5b) and up (Figure 5c), promoting the oxygen evolution (Figure 5b) and hydrogen evolution (Figure 5c) reactions, respectively. The relatively large value of the photocurrent measured is therefore not associated to the density of states, but to the large linear density of emerging non‐stoichiometric APBs (estimated to be 5 µm^−1^, from the linear APB density determined in Figure S15, Supporting Information, considering that the stoichiometry is preserved in average during all the propagation in the III–V layer, and that half of the emerging APBs correspond to stoichiometric configurations). Overall, the proposed mechanism explains why the bi‐domain III–V/Si material allows achieving ambipolar photoelectrochemistry. Moreover, the bi‐domain III–V samples grown on an n‐doped Si substrate show an anodic photocurrent higher than the cathodic one (Figure 2d–f) while the opposite situation is encountered for the sample grown on p‐doped Si with higher cathodic photocurrent (Figure S7, Supporting Information). This indicates that the local band bendings of Si with III–III and V–V APBs at the III–V/Si interfaces play a role in the process, as discussed in the Supporting Information (Figures S27–S30). In addition, the partial equilibration of the APB energy levels with the electrolyte at the solid/liquid interface may facilitate charge extraction in the electrolyte. This provides degrees of improvements to optimize charge carrier transfers, or even by making the Si substrate photoactive, with careful optimization of Fermi levels lineups through both III–V alloys composition or Si substrate doping. Therefore, the development of bidomain hybrid III–V/Si photoelectrodes not only gives the hope to reach the excellent performances of III–V photoelectrodes at a lower cost (due to earth abundancy of Si), and with simpler designs thanks to the internal built‐in electric field, but also opens up the possibility to push the efficiencies of PEC setups much beyond the ones of bare III–V photoelectrodes, through the III–V/Si tandem photoactive materials association. In addition, the ability to work both as a photoanode or photocathode allows considering new designs for integrated and compact PEC cells.

In summary, epitaxial bi‐domain III–V/Si comb‐like heterostructures with hybrid 2D semimetal/semiconductor properties (Figure 5d,e) enable not only photo‐generation through optical absorption and effective vertical ambipolar transport, but also promote efficient in‐plane charge carrier separation and charge transfers between bulk semiconducting domains and 2D semimetal singularities (Figure S31, Supporting Information). The Fermi level and bandgap engineering capabilities offered by the control of III–V ternary and quaternary alloys with today's epitaxial setups, the choice of Si substrate doping, and the possibility to tune the vertical or lateral APB distribution in III–V/Si samples,^[^ ^16^, ^26^ ^]^ are thus promising for the development of novel multifunctional photoelectric devices with bi‐domain III–V/Si materials. From the technical point‐of‐view, growing one hybrid bidomain III–V/Si sample is much easier than optimizing a whole p–n junction architecture, with issues of dopants incorporation and dopants activation. Additionnaly, p–n junction architectures used in photovoltaic or photonic architectures are usually 1–3 µm‐thick, which is not adapted for the development of compact and integrated devices, while hybrid III–V/Si are expected to generate internal built‐in electric fields as soon as APBs were formed (i.e., afer 10 nm deposited). Then, for computing or electronic applications, the anisotropy and localization of the current flow at the nanoscale, which can be transferred directly to the silicon chip, is of great interest, while the ability to manage local carriers injection and light emission offered by the bi‐domain hybrid materials could ease electrical injection in nano‐photonic devices. Finally, the ability of this hybrid material to absorb light and convert it into recoverable electrical current is definitely an advantage for solar energy harvesting, including PEC cells and photovoltaic architectures, but also more generally for sensors. In all these applications, it is important to consider that the density of the APBs may influence the performances. APBs density, and shape engineering and its impact on photoelectric properties would therefore be interesting to study for further device developments.

## 3 Conclusion

In conclusion, we demonstrated experimentally and theoretically that epitaxial bi‐domain III–V/Si heterostructures are hybrid and polyvalent materials, composed of bulk semiconducting domains enabling photo‐activity, and antiphase boundaries that are 2D topological vertical semi metallic inclusions enabling ambipolar transport properties. The demonstration of operating ambipolar III–V/Si photoelectrodes is completed by dedicated theoretical and experimental studies. From our data, it can be concluded that bi‐domain III–V/Si materials are indeed able within the same layer to absorb light efficiently, separate laterally the photo‐generated carriers, transfer them to semimetal singularities, and enable extraction of both electrons and holes vertically. Now that the ambipolar and photoactive behavior of these materials is understood and rationalized, further progress is expected to arise shortly by developing specific architectures for novel photoelectric devices. These multi‐functional properties obtained within the same layer seem to present many advantages for the future developement of energy harvesting, photonic, electronic or computing devices and may also expand a new route for the exploration of novel topological materials.

## 4 Experimental Section

### Sample Growth

The main bi‐domain III–V/Si samples (with GaP, GaPSb, and GaPAs in this work) were grown by molecular beam epitaxy (MBE) on HF‐chemically etched n‐doped (5 × 10^14–18^ cm^−3^), p‐doped (2.6 × 10^15^‐1.4 × 10^16^ cm^−3^), and high resistivity (>5000 Ω.cm) Si (001) substrates. The substrates were heated at 800 °C for 10 min to remove hydrogen at the surface. 1 µm‐thick III–V layers were then grown at 500 °C in a conventional continuous MBE growth mode, and at a growth rate of 0.24 mL s^−1^, with a beam equivalent pressure V/III ratio of 5. It should be noted that the whole epilayer was undoped, and epitaxial strategies to annihilate antiphase boundaries^[^ ^54^ ^]^ were not used here, leading to emerging APBs. The n‐doped GaP wafer used for comparison was 350 µm‐thick and the doping concentration was 10^17^–10^18^cm^−3^.

### Structural and Transport Characterizations

Chemical mechanical polishing was performed by using a 1% H_3_PO_4_ solution. The SEM images were obtained by using a JEOL JSM‐7100 scanning electron microscope. The atomic force microscopy (AFM) measurements were performed with a Veeco Innova AFM microscope equipped with a high‐resolution scanning probe. Tapping mode was used with the cantilever tuned around 293 kHz. Charge carrier type, density, and mobility were investigated by Hall effect measurements at room temperature. Samples were held in a 1.2 kOe magnetic field while sourced with a current in the 10–50 µA range leading to a power dissipation as low as 0.5 mW. Typical Hall voltages of tens of mV were measured with a high input impedance nanovoltmeter. The sheet resistance was also measured with the same setup using the classical Van Der Pauw geometry. Studied samples of 10 × 10 mm^2^ were directly contacted at the GaP surface by using osmium coated tungsten tips. The conductive atomic force microscopy (C‐AFM) measurements were carried with a Bruker Multimode AFM using a Pt/Ir coated Sb doped silicon cantilever (spring constant of 0.1 N m^−1^, and resonance frequency of 10 kHz). Before measurements, the sample surface was deoxidized using an HF 5% solution for 1 min, rinsed with ultra‐pure water, and then quickly transferred to AFM set‐up operated under dry nitrogen gas to limit humidity. A positive (+9V) or negative (−8.5V) bias was applied to the GaP/Si:n sample at 0.3 Hz in contact mode. All current mapping images were obtained with the cantilever moving across the thin film surface.

### Photoelectrochemical Measurements

The photoelectrochemical measurements were performed with a Zennium potentiostat (Zahner) in 1.0 m H_2_SO_4_ (pH = 0.3) electrolyte using a standard three‐electrode configuration with a graphite rod as the counter‐electrode and a KCl‐saturated calomel electrode Hg/Hg_2_Cl_2_ (SCE) as the reference electrode. The light was provided by a solar simulator (LS0106, LOT Quantum Design) equipped with an AM 1.5G filter. The measured potentials versus SCE were converted to the reversible hydrogen electrode (RHE) potentials using the following equation:

(1) $$E_{\mathrm{RHE}}=E_{\mathrm{SCE}}+0.24+0.059\;\mathrm{pH}$$

IPCE measurements were performed with a CIMPS‐QE IPCE 3 workstation (Zahner) comprising a TLS03 tunable light source controlled by a PP211 potentiostat in the same cell as the one used for classical electrochemical experiments and the applied potentials were set to −0.74 V and 1.26 V versus RHE. The light modulation frequency was 1 Hz, the settling time was 5 s, and the number of counts was 25. The Thales software provided the spectra in photocurrent efficiency (A W^−1^) or in IPCE (%). In order to check the validity of the IPCE measurements, the IPCE spectra were converted into photocurrent efficiency (in A W^−1^). Using these spectra and the AM 1.5G reference solar spectrum (American Society for Testing Materials http://rredc.nrel.gov/solar/spectra/am1.5/),^[^ ^55^ ^]^ the incident power was converted into a photocurrent spectrum that was integrated to obtain the theoretical value of photocurrent densities under AM 1.5G simulated sunlight.

The Mott–Schottky measurements were performed in the dark by sweeping the potential from positive to negative with an AC amplitude of 5 mV and 100 counts per point. The Mott–Schottky equation relates the capacitance *C* to the applied potential *E* across a semiconductor–electrolyte junction following:

(2) $$\frac{1}{C^{2}}=\frac{2}{eN_{\mathrm{Dopant}}A^{2}\varepsilon_{0}\varepsilon_{r}}\;\left(E-E_{\mathrm{fb}}-\frac{kT}{e}\right)$$

where, *ε*_r_ is the relative permittivity of the semiconductor, *ε*_0_ is the permittivity in vacuum, *A* is the surface area, *e* is the charge of an electron, *N*_Dopant_ is the free carrier density, *k* is the Boltzmann constant, and *T* is the temperature. *E*_fb_ corresponds to the flatband potential which can be extracted from the *x*‐intercept of the linear portion of the MS curve.

The electrochemical impedance spectroscopy (EIS) measurements were performed at three different potentials: open circuit potential (OCP) (0.16 V vs RHE for dark state and −0.24 V vs RHE for light state), anodic side (OCP + 0.5 V) (i.e., 0.66 V vs RHE for dark state and 0.26 V vs RHE for light state) and cathodic side (OCP−0.7 V) (i.e., −0.54 V vs RHE for dark state and −0.94 V vs RHE for light state). The frequency was varied from 100 kHz to 1 Hz with an AC amplitude of 5 mV.

### First‐Principles Calculations

For the slab structures constructions, thick vacuum regions were added on both sides (≈20 and 30 Å vacuum regions for the short and long slab structures) to avoid any interaction between slab surfaces. Band structure calculations were performed with the density functional theory (DFT)^[^ ^56^ ^]^ as implemented in the Vienna ab initio simulation package^[^ ^57^, ^58^ ^]^ with the projector augmented‐wave method,^[^ ^59^, ^60^ ^]^ based on Heyd–Scuseria–Ernzerhof (HSE) hybrid^[^ ^61^, ^62^, ^63^ ^]^ and metaGGA (TB‐mBJ)^[^ ^45^ ^]^ functionals with an energy cutoff of 500 eV. Besides, topology related (including band structures and Berry phase) calculations were carried out by generating ab initio tight‐binding Hamiltonians from the maximally localized Wannier functions within HSE potential,^[^ ^42^ ^]^ as implemented in Wannier Tools package.^[^ ^64^ ^]^ The Bloch wave functions are projected onto the s, p, d atomic orbitals of Ga and s, p atomic orbitals of P, As, and Sb. The standard generalized gradient approximation (GGA) parameterized by Perdew–Burke–Ernzerhof (PBE)^[^ ^65^ ^]^ was used for structure optimization and the structures were relaxed until the Hellmann–Feynman forces on the atoms were less than 10^−4^ eV Å^−1^. The symmetry properties of the electronic states were analyzed starting from the irreducible representations provided by Quantum Espresso (QE) package codes.^[^ ^66^, ^67^ ^]^

### Statistical Analysis

The present study did not require any specific statistical analysis tools.

## Conflict of Interest

The authors declare no conflict of interest.

## Author Contributions

C.C. conceived the idea. C.C., N.B., R.B., and T.R. designed and fabricated the samples. J.L.‐P. carried out the SEM experiments. L.C. performed AFM measurements. R.B. made the C‐AFM measurements. S.T., P.T., and P.S. did and analyzed the Hall measurements. L.C., G.L., and M. P. performed the PEC measurements and L.C., Y.L., G.L., N.B., B.F., and C.C. analyzed the corresponding results. A.B. fabricated the GaP nanomembranes. C.C. and C.L. contributed to structural investigations analysis. L.C., I.J., Y.L, N.B., and C.C. contributed to the analysis of ambipolar transport properties. L.C., J.E., and L.P. performed and analyzed the DFT calculations. J.E. and L.C. performed the symmetry analysis. L.C., J.E., Y.L. C.C., and L.P. contributed to topological property analysis. L.C. and C.C. wrote and finalized the manuscript. All authors reviewed and commented on the manuscript.

## Supporting information

Supporting Information

## Data Availability

Research data are not shared.
