# Supplementary material for: Epitaxial III–V/Si Vertical Heterostructures with Hybrid 2D‐Semimetal/Semiconductor Ambipolar and Photoactive Properties
Source: Adv Sci (Weinh). 2021 Nov 11;9(2):2101661. doi: 10.1002/advs.202101661 (PMC8805590; doi:10.1002/advs.202101661)
Supplement: Supplementary file 1 — Supporting Information [file ADVS-9-2101661-s001.pdf]

© 2021 Wiley-VCH GmbH

ADVANCED SCIENCE
Open Access

Supporting Information

for *Adv. Sci.,* DOI: 10.1002/advs.202101661

Epitaxial III-V/Si vertical heterostructures with hybrid 2D-semimetal/semiconductor ambipolar and photoactive properties

*Lipin Chen, Yoan Léger, Gabriel Loget, Mekan Piriyev, Imen Jadli, Sylvain Tricot, Tony Rohel, Rozenn Bernard, Alexandre Beck, Julie Le-Pouliquen, Pascal Turban, Philippe Schieffer, Christophe Levallois, Bruno Fabre, Laurent Pedesseau, Jacky Even, Nicolas Bertru and Charles Cornet**

Supporting Information

Epitaxial III-V/Si vertical heterostructures with hybrid 2D-semimetal/semiconductor ambipolar and photoactive properties

*Lipin Chen, Yoan Léger, Gabriel Loget, Mekan Piriyev, Imen Jadli, Sylvain Tricot, Tony Rohel, Rozenn Bernard, Alexandre Beck, Julie Le-Pouliquen, Pascal Turban, Philippe Schieffer, Christophe Levallois, Bruno Fabre, Laurent Pedesseau, Jacky Even, Nicolas Bertru and Charles Cornet**

Atomic structure of a stoichiometric APB

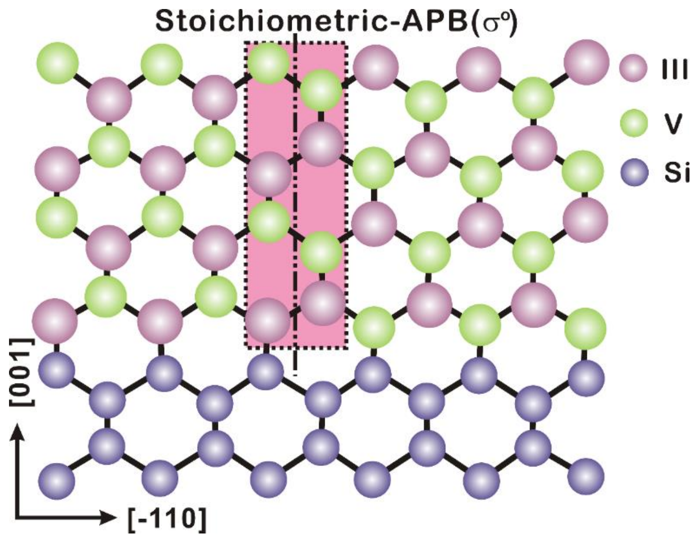

Figure S1: Schematic of the stoichiometric-APB atomic configuration.

The atomic configuration of a stoichiometric APB with equal numbers of III-III and V-V bonds within the same APB is illustrated in Figure S1.[1]

Scanning Electron Microscopy (SEM) images and APB developing treatment

The III-V/Si samples presented in this work are 1 µm-thick, and thus the distribution of APDs cannot be characterized by using the conventional X-ray Diffraction Williamson-Hall approach.[2] A specific APB developing treatment combining Chemical Mechanical Polishing (CMP) and SEM was thus used to evidence and characterize the APB distribution.[3]

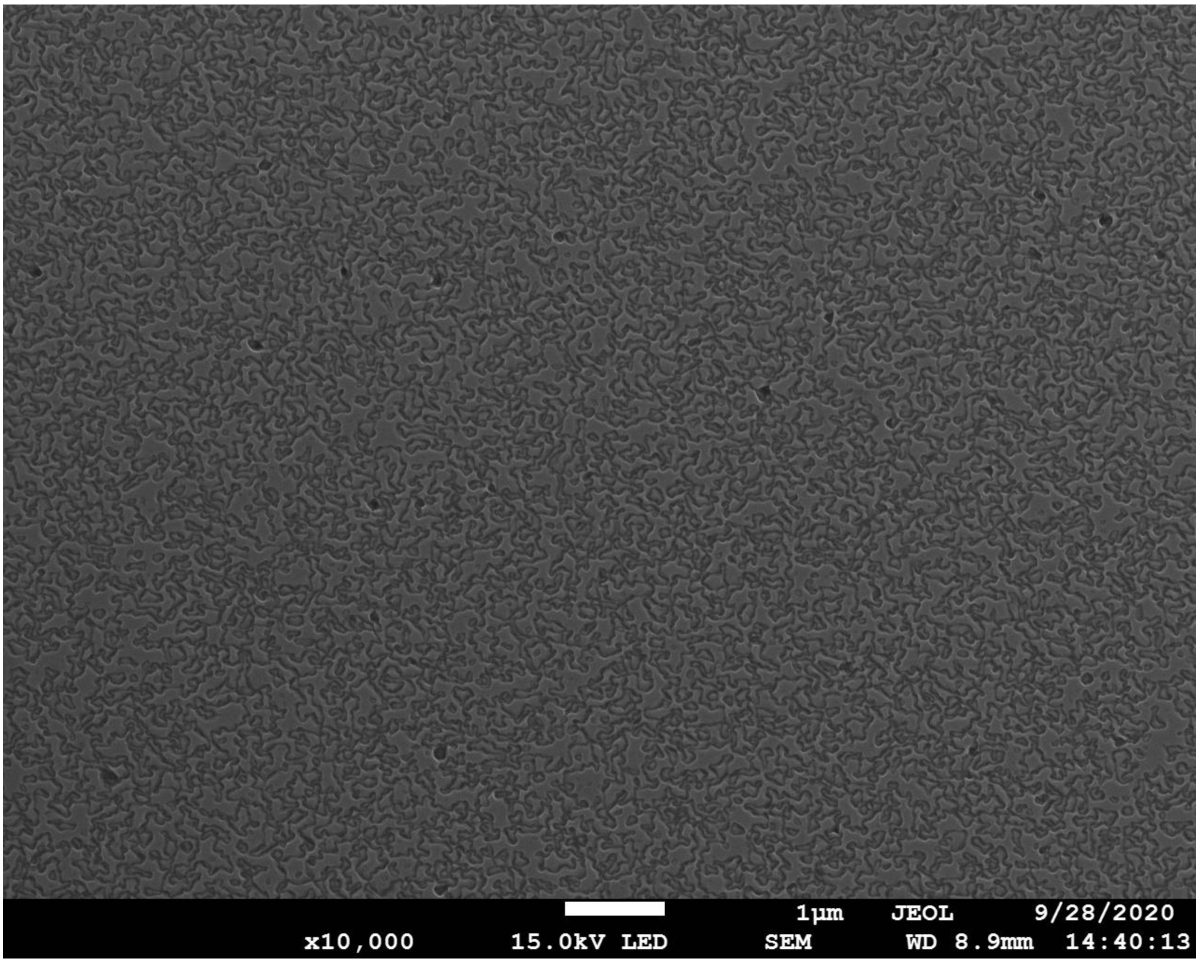

Figure S2a: 12 x 9 µm² Plane-view SEM image of the bi-domain GaP/Si sample used for Photo-electrochemical characterizations in this work after APB developing treatment at the surface. APBs are evidenced as noodles-like structures at the surface.

The sample surface (usually rough for thick III-V/Si samples with APBs, rms > 10 nm) is first polished with H_3PO_4 chemical-mechanical (CMP) polishing, to retrieve a flat surface (rms roughness below 1 nm). From this flat surface, the sample is then chemically etched using the GaP-etch commercial solution. With this chemical solution, the etching rate is

strongly affected by the presence of APBs.[4] After a careful calibration of etching rates, it becomes possible to reveal only emerging APBs in the sample, in the form of roughness induced at the surface during the etching process, as shown in Fig. S2a. From this picture, one cannot only obtain the spatial distribution of emerging antiphase domains with a remarkable accuracy on samples surface as large as 10 x 10 µm² independently of the roughness induced by other defects or faceting effects, but also use binarized images and Fourier analysis to extract all the statistical information (about correlation length, average polarity, and so on…) from more than 100 domains identified. The x10,000 SEM image shown below, performed after that the SEM+CMP process was applied on the 1µm-thick GaP/Si sample used in the article, reveals the homogeneity of the APD distribution over a very large area of 12 x 9 µm² and we confirm that such an APD distribution is observed on the whole surface of the coupons of a few square mm used for PEC measurements and other measurements.

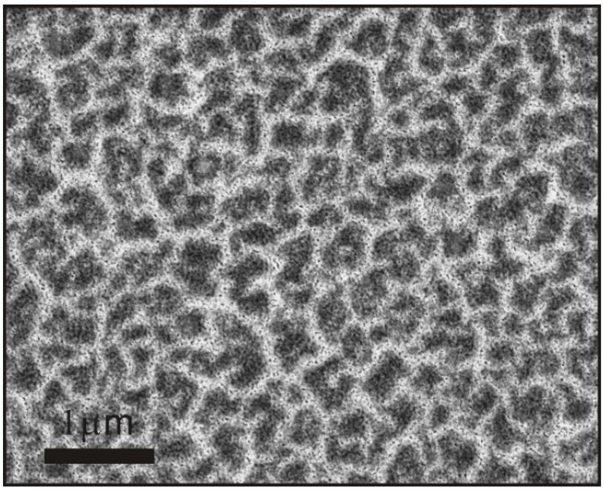

Figure S2b: Plan-view SEM image of a bi-domain GaPSb/Si sample after the APB developing treatment. The white lines correspond to APBs, due to the faster chemical etching of APBs.

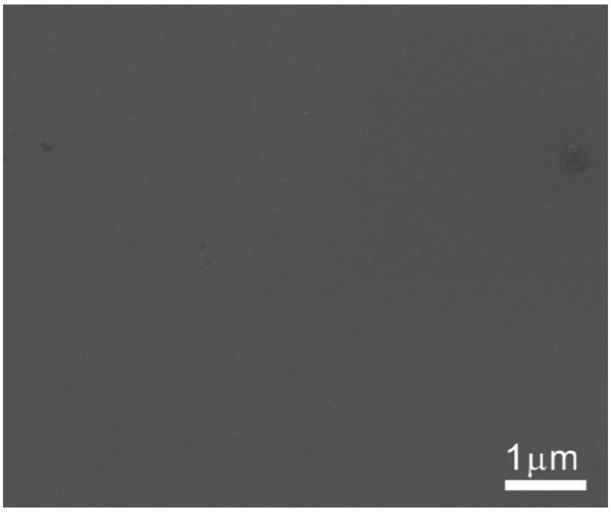

Figure S3: Plan-view SEM image of a n-doped GaP wafer.

Figure S2b shows the plan-view SEM image of one bi-domain GaPSb/Si sample after the APB developing treatment, where the APBs emerging at the surface are shown with a denser and random honeycomb-like lattice. Figure S3 shows a typical plan-view SEM image of a n-doped GaP wafer with a clean surface observed under the same conditions as for Figure S2.

Fabrication of bonded GaP nanomembranes

For the fabrication of bonded GaP nanomembranes from GaP/Si:n samples, firstly a Si host substrate with a 1μm-thick SiO_2 layer obtained by thermal oxidation at the surface, was prepared. A 200 nm-thick silicon nitride layer was then sputtered on the GaP/Si MBE samples to improve adhesion of benzocyclobutene (BCB). Host substrate and GaP/Si samples were then bonded together using BCB as an adhesive layer. BCB was deposited on the host substrate by spin-coating. The BCB deposition is followed by a soft bake at 140°C on a hotplate for 2 minutes. Meanwhile, the GaP/Si is also kept at this temperature on the same hotplate. Both GaP/Si sample and host substrate were then removed from the hotplate, put in contact together and placed back on the hotplate for an additional soft bake of 2 minutes. During this additional soft bake, a gentle pressure is applied manually. Then, the bonded sample was hard baked at 250°C under nitrogen flow for 1 hour. Following bonding, the

growth silicon substrate was removed in a hot 5% TMAH bath (Sigma). The etch rate of silicon is about 1 μm/minute and etching naturally stops on the GaP layer.

Hall measurements

Estimation of the parallel to perpendicular resistivity ratio. Let us call $R_{\parallel}$ ($R_{\perp}$) the total in-plane (out-of-plane) APBs resistance in the GaP layer and $\rho_{\parallel}$ ($\rho_{\perp}$) the corresponding resistivity. We have:

$$\frac{\rho_{\parallel}}{\rho_{\perp}} = \frac{R_{\parallel} \times \alpha_{\perp}}{R_{\perp} \times \alpha_{\parallel}} > \frac{R_{\parallel,\mathrm{min}}}{R_{\perp,\mathrm{max}}} \times \frac{\alpha_{\perp}}{\alpha_{\parallel}}$$

$\alpha_{\parallel,\perp}$ are geometrical coefficients describing the volume probed by charge carriers due to the electric field extension in the sample between the contacts. This resistivity ratio is minimal for the low limit value of $R_{\parallel}$ ($R_{\parallel,\mathrm{min}}$) and for the high limit value of $R_{\perp}$ ($R_{\perp,\mathrm{max}}$). $R_{\parallel,\mathrm{min}}$ is evaluated from GaP with APBs membrane. As it was impossible to detect any current, we deduced that the true resistance is at least higher than the voltmeter input impedance (namely 10 GΩ). $R_{\perp,\mathrm{max}}$ was measured from the current-voltage curve shown in Fig. S4, for 2 contacts on top of the GaP/Si-n sample. The inset in Fig. S4 displays the experimental setup used to perform electrical measurements. The total resistance R is the slope extracted from a linear fit to this curve. It is the sum of (i) contact resistances lying at the tip/GaP interface (R_{c_1}) and at the buried GaP/Si interface (R_{c_2}) as well as (ii) the resistance of the GaP layer under the tips ($R_{\perp}$) and finally (iii) the resistance of the silicon substrate (R_{Si}). Thus, the total resistance is:

$$R = 2 \times \left(R_{c_1} + R_{c_2} + R_{\perp}\right) + R_{\mathrm{Si}} = 6.4\ k\Omega$$

$R_{\perp,\mathrm{max}}$ was obtained by assuming that contact resistances were negligible compared to $R_{\perp}$and by taking R_{Si} equal to the sheet resistance of the substrate (170 Ω) which minimizes the silicon resistance by assuming that all the silicon volume was probed by charge carriers. We obtained a maximum value for the out-of-plane resistance $R_{\perp,\mathrm{max}}$ around 3.2 $k\Omega$.

For the in-plane resistance, the geometrical factor is $\alpha_{\parallel} = L/(W \times t)$, where L is the distance between the contacts, t is the thickness of the GaP membrane and W is the width of the cross-section the current is flowing through. Probes are close to the center of the sample and edges of the sample are located further than 5 times the probe spacing. In such conditions, the electric field lines extend as wide as the spacing between the probes, thus $L \approx W$.

For the perpendicular resistance, the geometrical factor is $\alpha_{\perp} = \frac{t}{\pi r^2}$, with r being the radius of the contact probe, r is typically of 15 μm, and t the thickness of the GaP layer. We find eventually,

$$\frac{\rho_{\parallel}}{\rho_{\perp}} > \frac{R_{\parallel,\min}}{R_{\perp,\max}} \times \frac{t^2}{\pi r^2} = 3.2 \times 10^3$$

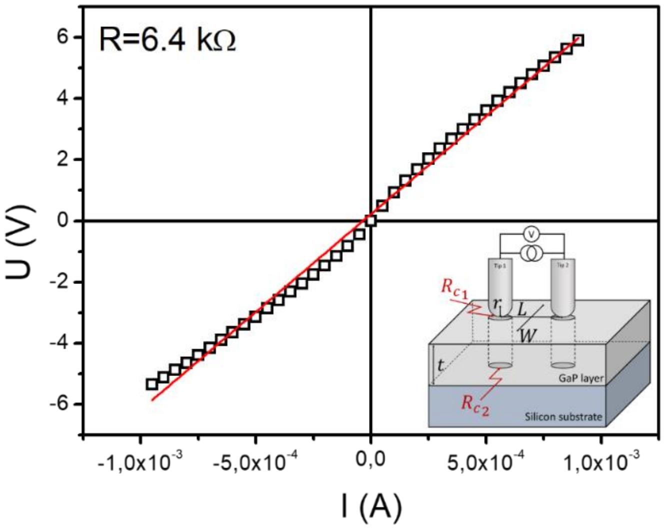

Figure S4: Typical two-probe voltage vs current curve for GaP (with APBs) on Si-n substrate.

The obtained resistivity ratio clearly demonstrates that GaP layers exhibit a large parallel to perpendicular conductivity anisotropy. We think that the perpendicular resistance extracted from our measurements is probably dominated by the tip/GaP layer contact resistance and by the Shottky barrier that may exist at the GaP/Si interface. The value of the latter could be estimated from temperature dependent measurements.

Conductive atomic force microscopy (C-AFM) images for bi-domain III-V/Si samples

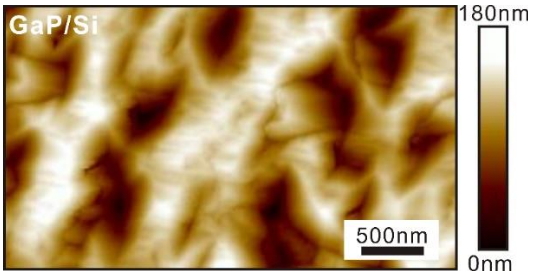

Figure S5: Topography of the bi-domain GaP/Si sample as measured with the conductive atomic force microscopy (C-AFM) experimental setup.

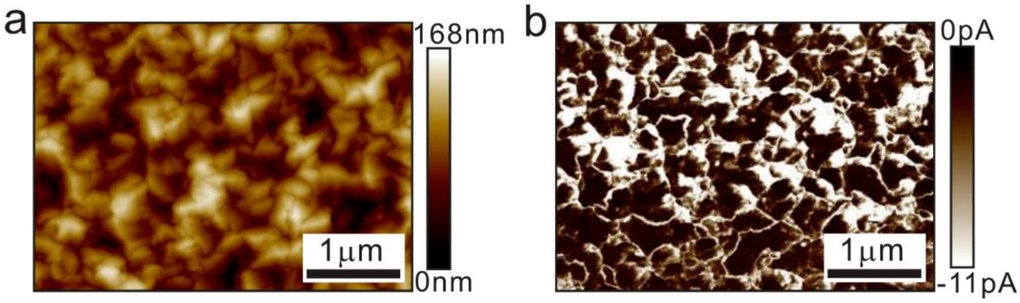

Figure S6**:** Topography (a) and current distribution (b) C-AFM images (DC bias = -7.5V) of the bi-domain GaPSb/Si sample.

Figure S5 shows the topography of the bi-domain GaP/Si:n sample, as measured with the C-AFM experimental setup, which corresponds to the current distribution image in Fig. 2a and Fig. 2b. Figure S6 shows the topography and current distribution images of the bi-domain GaPSb/Si:n sample under negative bias (-7.5V), which reveals the good conductivity of the APB singularities.

Chopped-light linear sweep voltammetry curves of GaPSb sample on p-doped Si substrate

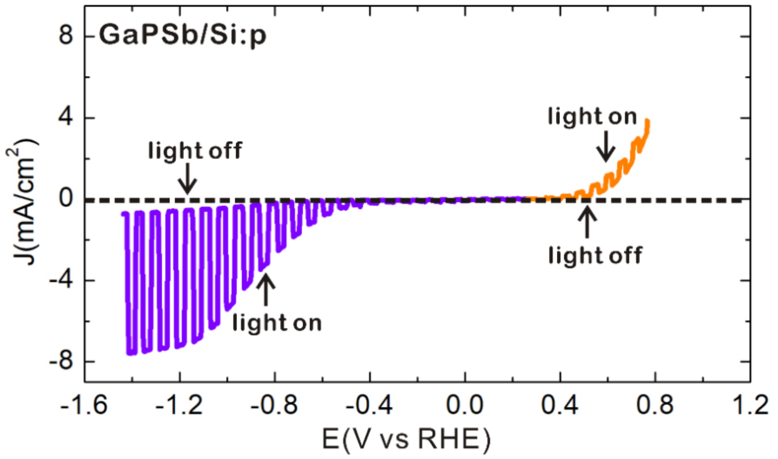

Figure S7: Voltammetry curve of the bi-domain GaPSb/Si:p in 1.0M H_2SO_4 (pH=0.3) electrolyte under chopped simulated sunlight.

Figure S7 shows the voltammetry curve of the bi-domain GaPSb layer on p-doped Si substrate, revealing the ambipolar properties. Besides, the cathodic photocurrent is higher than the anodic one, which is the reverse behavior to the trend observed for the bi-domain III-V sample on n-doped Si substrate.

Mott-Schottky (MS) plots of GaP:n wafer

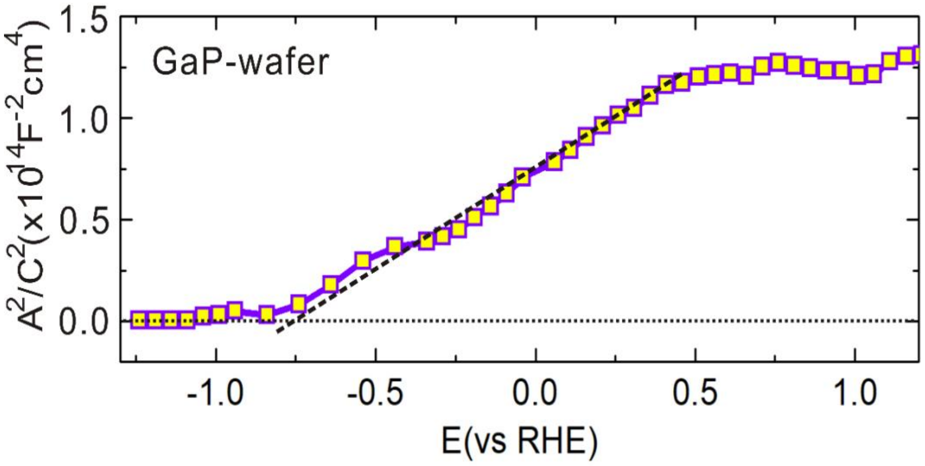

Figure S8: MS plot recorded at 500 Hz for GaP wafer.

The Mott-Schottky (MS) plot (Fig. S8) of the GaP:n wafer shows a positive slope demonstrating the n-type character. From the slope, a donor density of 2×10^{17} cm^{-3} is calculated which is in close agreement with the specification of the GaP wafer.

Stoichiometric APB structure for DFT band structure calculation

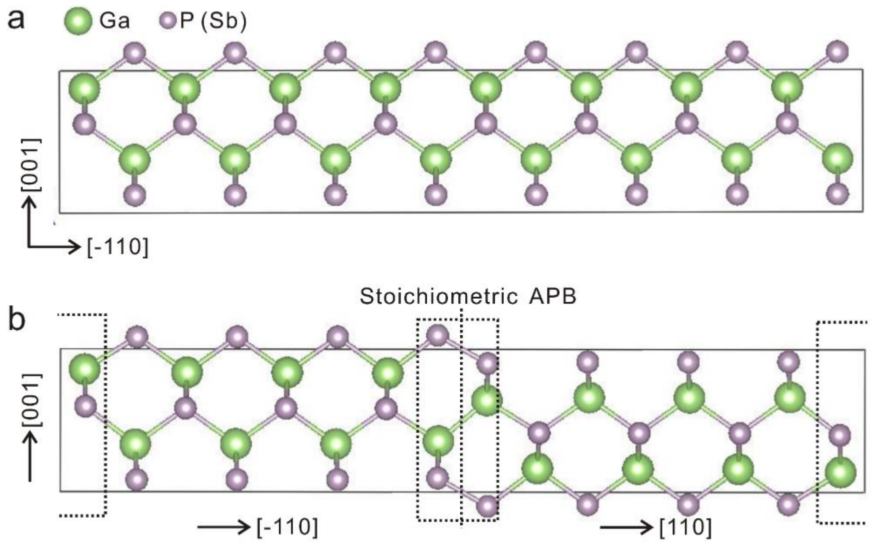

Figure S9: III-V bulk-supercell (a) and stoichiometric APB (b) structures for band structure calculations.

Fig. S9b shows the stoichiometric APB structures (called hereafter stoichiometric APB supercell) for band structure calculations. For comparison, the corresponding calculations were also done for supercell structures without APB (called hereafter bulk-supercell) (Fig. S9a).[1] The corresponding calculation results are shown in Fig. S24 and Fig. S25.

DFT band structures based on HSE potential for a zinc blende unit cell of GaP, GaAs and GaSb

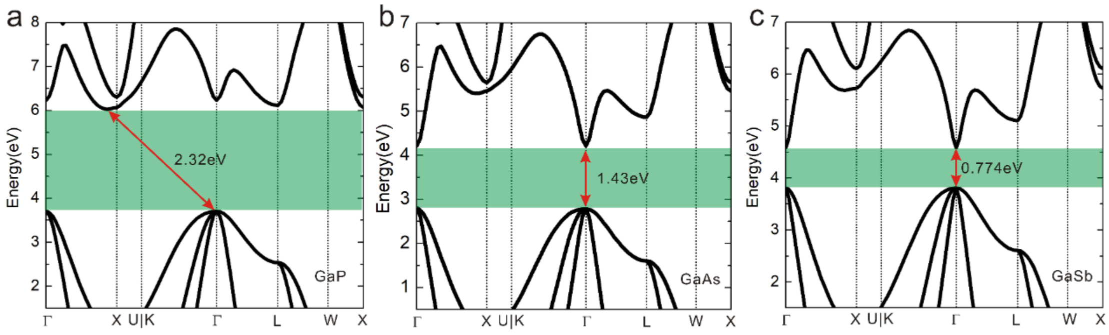

Figure S10: DFT band structures based on HSE potential for a zinc blende unit cell of GaP (a), GaAs (b) and GaSb (c).

DFT band structures based on Heyd–Scuseria–Ernzerhof (HSE) hybrid functional were performed for the zinc blende unit cell structure of GaP (Fig. S10a), GaAs (Fig. S10b) and GaSb (Fig. S10c), showing respective bandgap of 2.32eV, 1.43eV and 0.774eV.

Longer non-stoichiometric APB slab structures

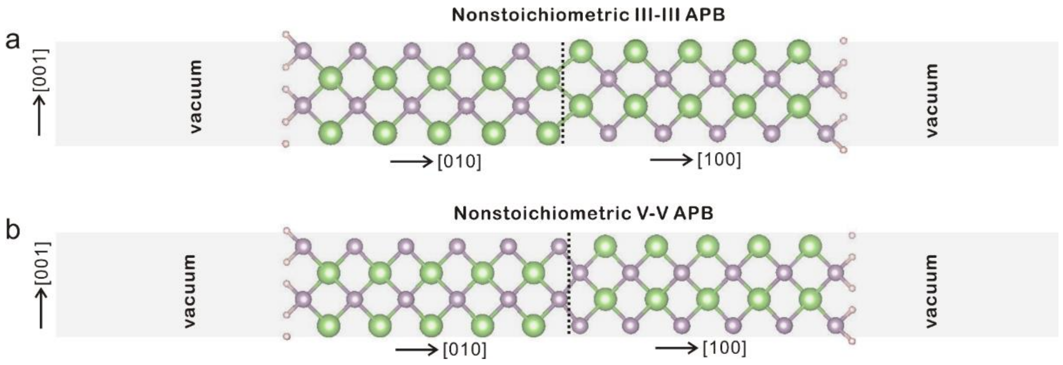

Figure S11: Longer III-III (a) and V-V (b) APB slab structures.

Figure S11 shows the longer nonstoichiometric III-III (Fig. S11a) and V-V (Fig. S11b) APB slab structures used for charge density (Figure 3 m,n) and Fermi energy calculations (Figure 4 m, n, o).

Little co-groups for various k-points in the reciprocal space related to the nonstoichiometric APB structures

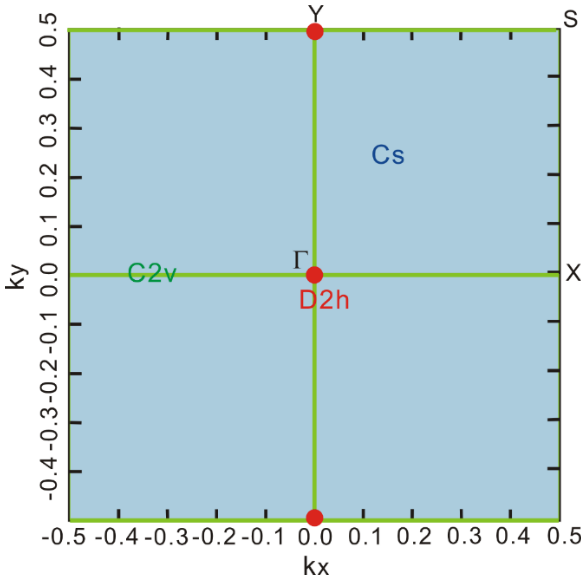

Figure S12: Symmetry properties of various k-points related to III-III and V-V non-stoichiometric APB structures with Pmma space group.

In the reciprocal space of the non-stoichiometric APB structures, the high-symmetry points of Γ, X, Y, and S correspond to the D2h point group (the red points in Fig. S12), the points on the lines connecting high-symmetry points to the C2v point group (the points on the green lines in Fig. S12), and the other k points to the Cs point group (the points in the blue region in Fig. S12).

Electronic bands of nonstoichiometric APB structures based on first-principle tight-binding model

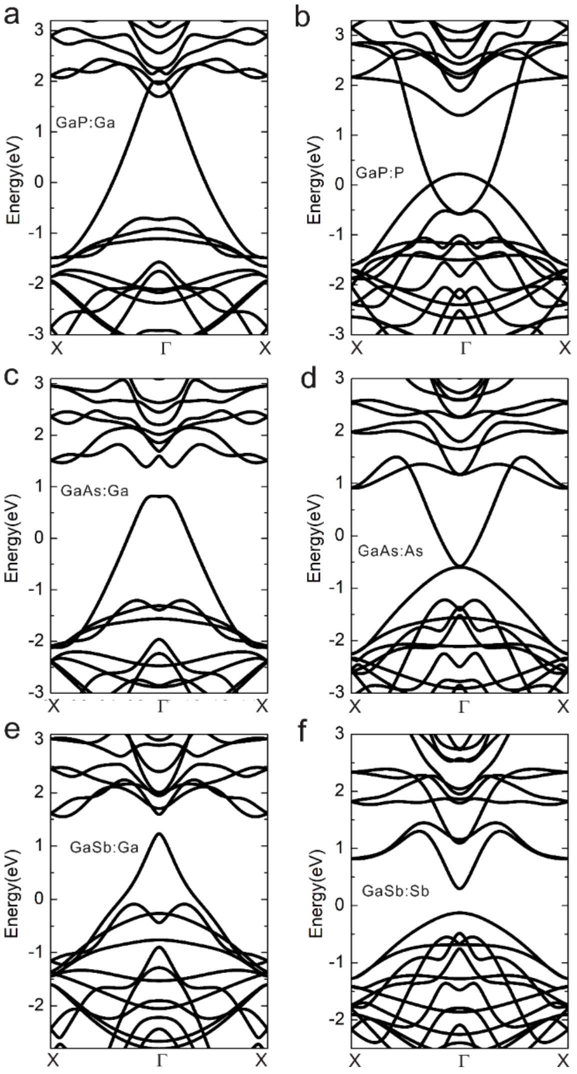

Figure S13: Band structures along X-Γ-X based on first-principle tight-binding model for GaP (a,b) GaAs (c,d) and GaSb (e,f)

The band structures along k path X- Γ-X of III-III and V-V APB structures based on GaP, GaAs and GaSb are extracted from first-principle tight-binding model (as shown in Fig. S13), which show very good consistency with those extracted from DFT calculations based on HSE potential (Fig. 3), verifying the accuracy of the calculations.

Realistic density of states extracted from the total density of states of nonstoichiometric APB slab structures and experimental linear density of APBs

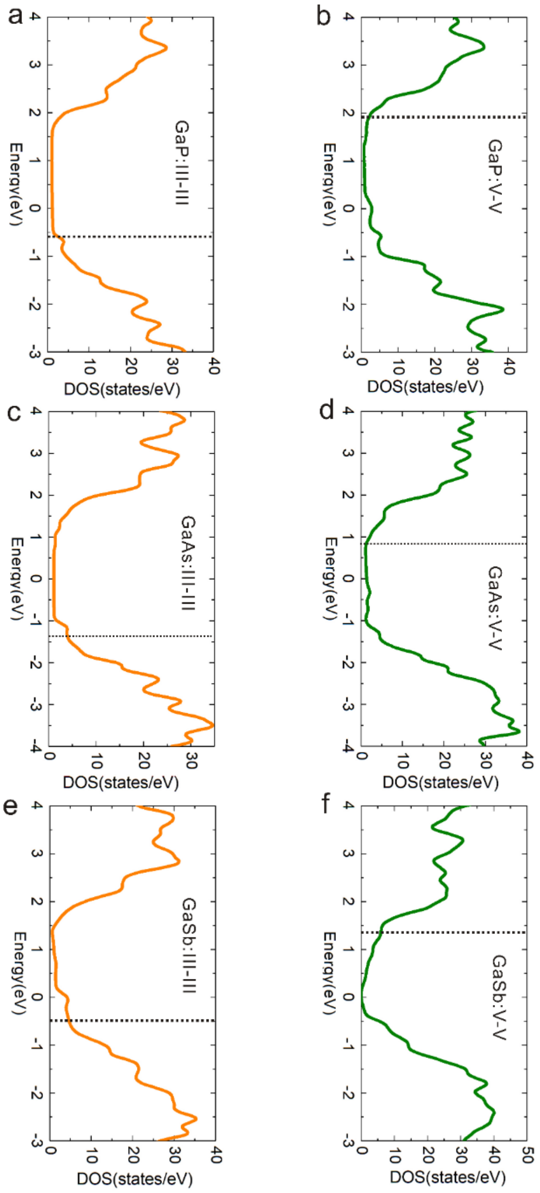

Figure S14: Total density of states of the III-III and V-V APB slab structures for GaP (a,b), GaAs (c,d) and GaSb (e,f) based on first-principle tight-binding model.

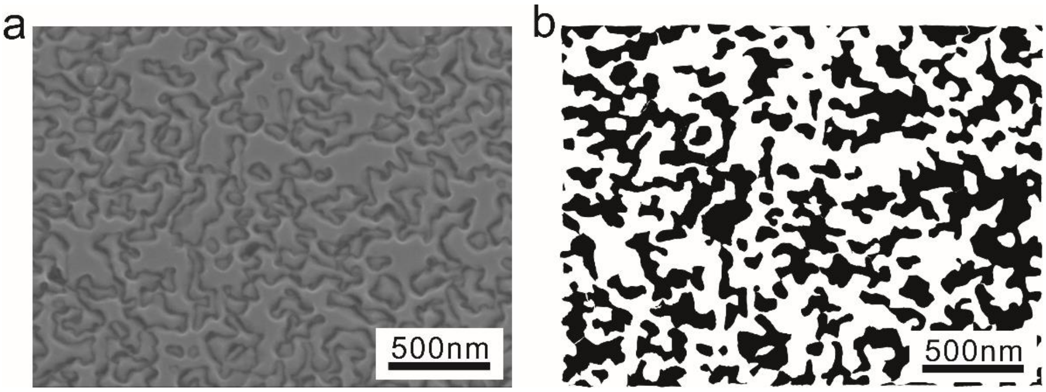

Figure S15: Original (a) and binarized (b) SEM images for the estimation of linear density of APB

Figure S14 shows the total density of states (units: states/eV) of the III-III and V-V APB slab structures (Fig. 3b,c) for GaP (a,b), GaAs (c,d) and GaSb (e,f), extracted from first-principle tight-binding model. What should be emphasized is that the total density of states corresponds to the slab structures, which does not necessarily match the properties of the real material. As the density of states is the superposition of APB density of states and bulk-like densities of states, it is necessary to consider that the experimental linear density of APBs is in fact smaller than the ones considered for the simulation because the sizes of the supercells are limited by the computational resources. In the real material, the linear density of APB was estimated to 10.47 μm^{-1}, from the SEM image (Fig. S15), i.e. around 1 APB each 100 nm of bulk. While, the linear density of APB of the supercell for calculation, taking III-III APB slab structure for example, is 0.5826nm^{-1} i.e. around 1 APB each 1.7163 nm of bulk. Therefore, the APB part of the total density states of the III-III APB supercell (Fig. S14a) remains unchanged (i.e. multiplied by 1) and the bulk part needs to be multiplied by 58.26 (100nm/1.71631nm) to get a more realistic total density of states. Finally, the total density of states is divided by the volume and we can get a realistic density of states expressed in states/eV/μm^3 unit for the III-III APB structure corresponding to structural investigations, as shown in Fig. 4b. In the same way, the density of states for the other III-III and V-V APB structures can be estimated, and are shown in Fig. 4 d,f,h,g,l.

DFT band structure calculations of nonstoichiometric APB structures based on metaGGA (TB-mBJ) potential: analysis of the crossing points

3D band structure plots of non-stoichiometric APB structures extracted from first-principle tight-binding model (Fig. 4) show that the crossing points exist in the form of pairs of discrete points (called Weyl point[4]) and along special lines creating either closed rings (called nodal ring[3]) or non-closed lines (called nodal line[5]) in momentum space. In order to further

analyze the crossing points, DFT electronic band calculations of nonstoichiometric APB structures for GaP based on metaGGA (TB-mBJ) potential are performed along multiple k paths with dense k points together with band symmetry analysis.

Fig. S16 displays the selected k-paths in the k_z=0 plane for electronic band calculations for the III-III APB structure centering around Γ and Y points. The corresponding band structures and enlarged band structures centering around Γ points are shown in Fig. S17 and Fig. S18, where the irreducible representations (IRs) of the CBM and VBM are indicated. We can observe that the bands open gaps around 40-60 meV (Fig. S18) due to the hybridization of the same irreducible representation of the CBM and VBM.[5] The band structures and enlarged band structures centering around Y points are shown in Fig. S19 and Fig. S20, together with the IRs of the CBM and VBM around crossing points. The different IRs of the CBM and VBM avoid hybridization effects and protect the crossing points, which are evidenced by the enlarged band structures (Fig. S20). The crossing points are marked by green dots in the k-path illustration (Fig. S16), which form one pair of Weyl points and one nodal ring centering around Y point, showing good agreement with the 3D plot band structure based on first-principle tight-binding model (Fig. 4a). In the same way, the electronic band calculations for the V-V APB structure are also performed along multiple k paths at k_z=0 plane (as shown in Fig. S21). The band structures together with IRs of the CBM and VBM are shown in Fig. S22 and Fig. S23, which indicate the existence of band crossing. The crossing points marked in Fig. S21 as purple dots form two snake-like nodal lines, which shows good consistency with the Fig. 4c.

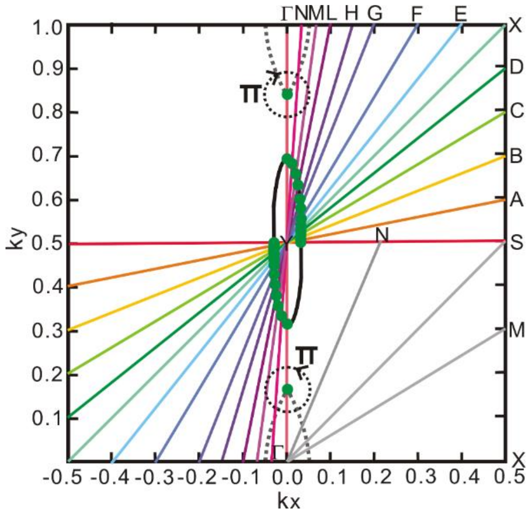

Figure S16: Illustration of selected k-paths in the k_z=0 plane for band structure calculations with the III-III APB structure. Crossing points are marked by green dots.

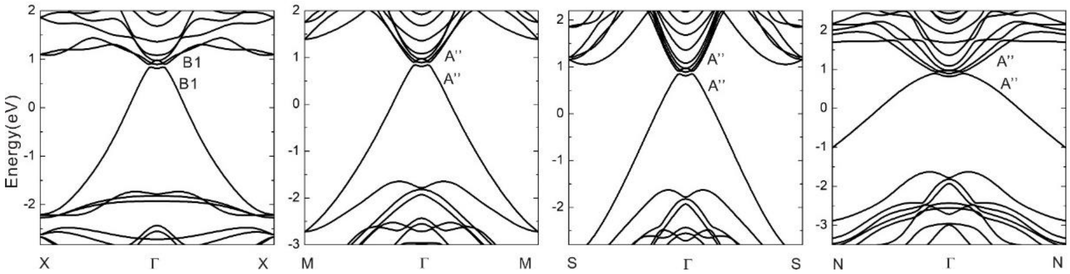

Figure S17: Band structures with band symmetry information of III-III APB structure along k-path X-Γ-X, M-Γ-M, S-Γ-S and N-Γ-N.

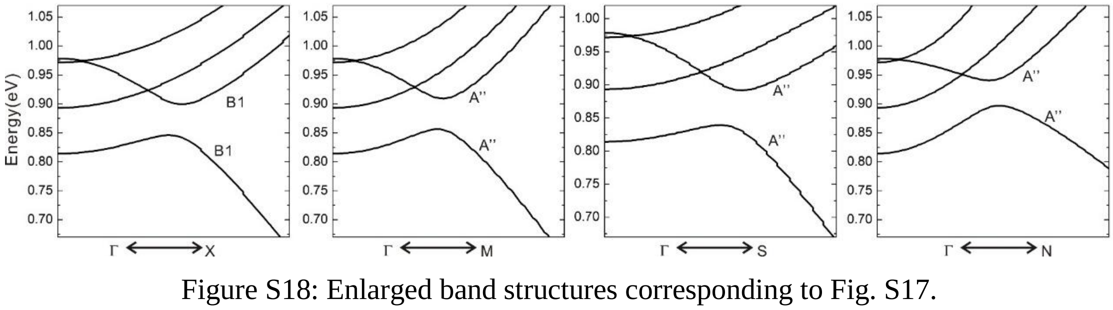

Figure S18: Enlarged band structures corresponding to Fig. S17.

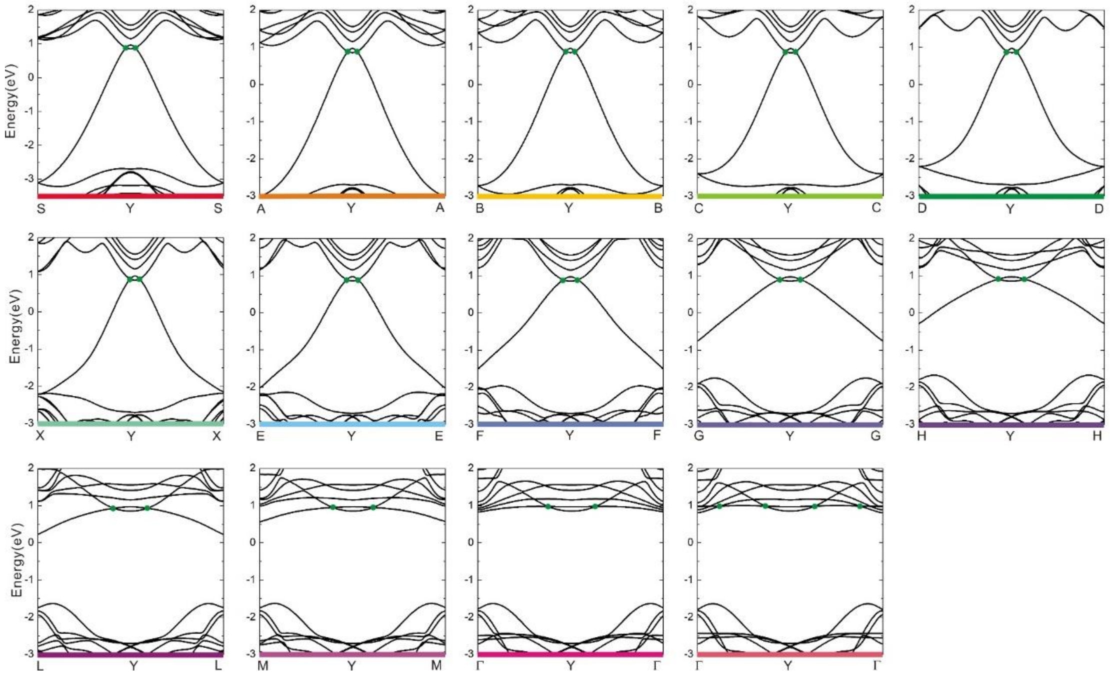

Figure S19: Band structures with band symmetry information of III-III APB structure along the different k-paths around the Y high symmetry k point.

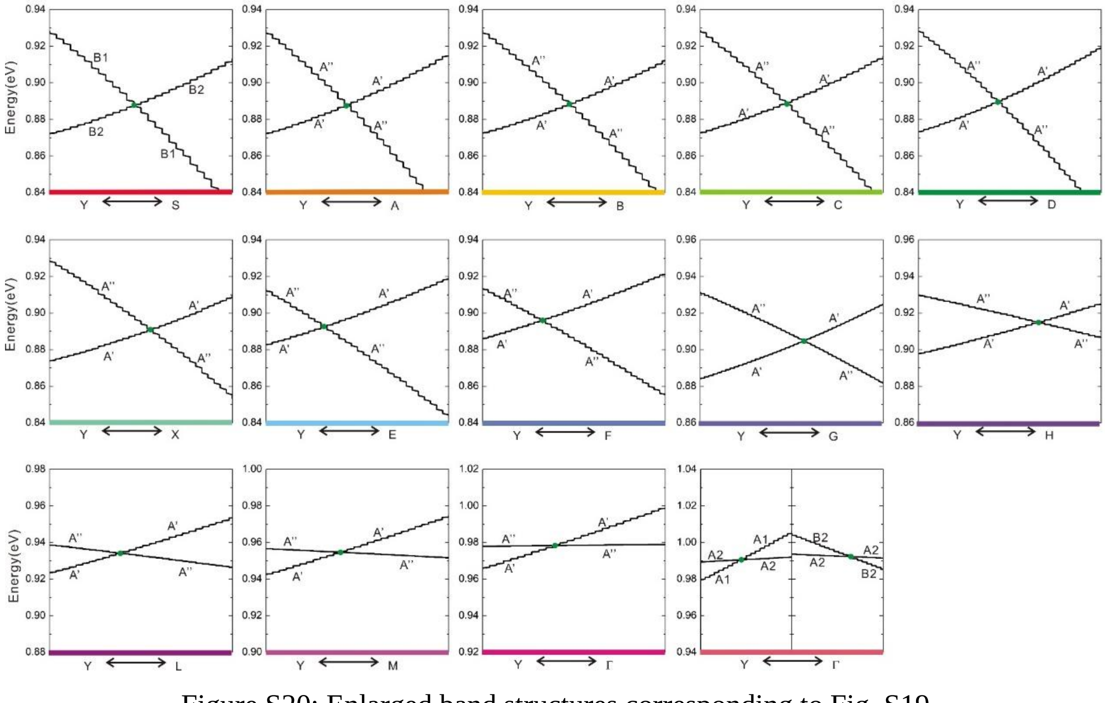

Figure S20: Enlarged band structures corresponding to Fig. S19.

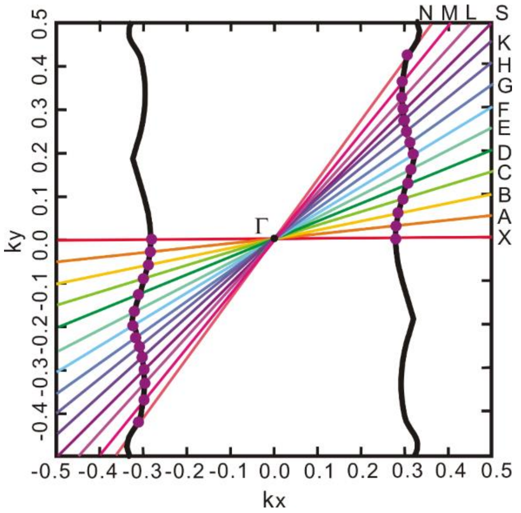

Figure S21: Illustration of selected k-paths in the k_z=0 plane for band structure calculations based on V-V APB structure and the crossing points marked by purple dots.

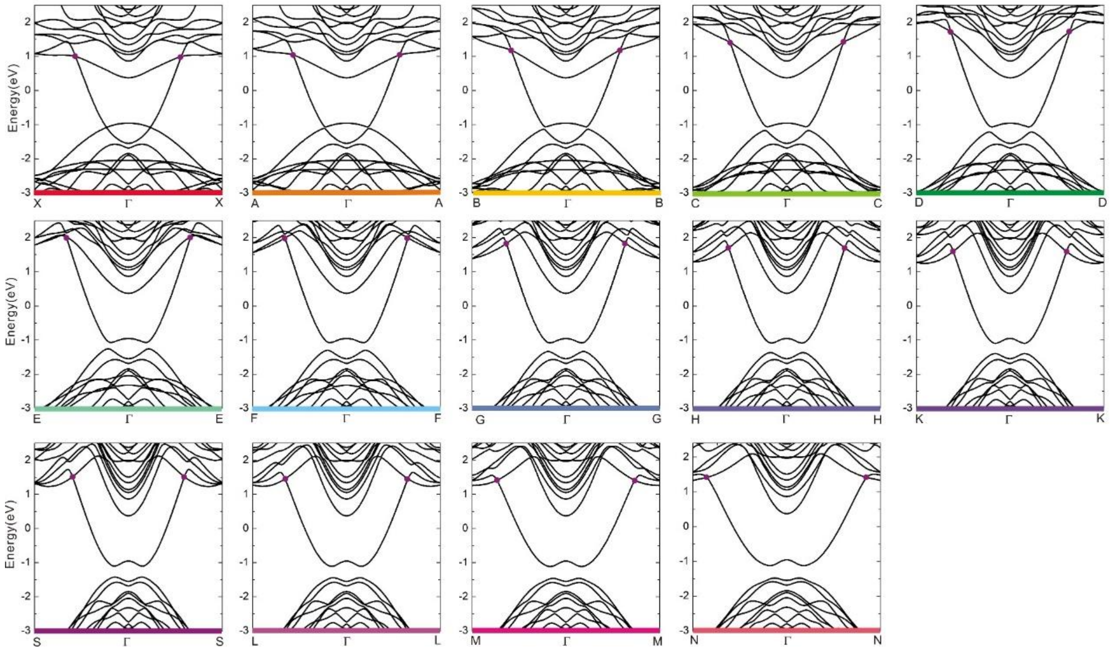

Figure S22: Band structure with band symmetry information of V-V APB structure along the different k-paths around the Γ high symmetry k point.

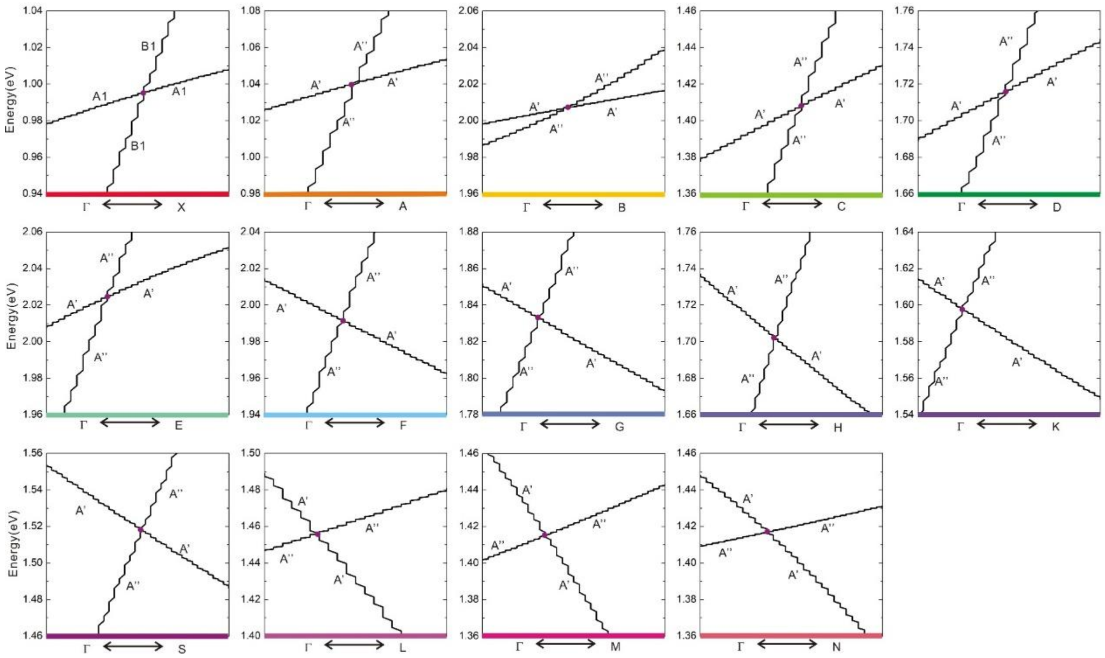

Figure S23: Enlarged band structures corresponding to Fig. S22.

Additional DFT calculations on stoichiometric APB structures

Additional calculations of band structures were also performed by DFT for stoichiometric APB supercell (Fig. S9b) for GaP and GaSb. For comparison, the corresponding calculations were also done for GaP and GaSb bulk-supercell (Fig. S9a).[1] Electronic bands were calculated along different k-paths (Z(0,0,0.5))-Γ(0,0,0)-U(0.5,0,0.5); R(0.5,0.5,0.5)-Γ(0,0,0)-S(0.5,0,0.5)), where the axes x, y and z of the reciprocal space correspond to the [001], [1-10], [110] crystallographic directions, respectively, based on Heyd–Scuseria–Ernzerhof (HSE) hybrid functional. The band structure plots of the stoichiometric APB structure with GaP and GaSb are shown in Fig. S25a and Fig. S25b, respectively, where the states are weighted by their spatial localization at the APB plane. The color map from blue to red underlines the increase of localization effects of the states at the APB atoms. Figure S24 shows the band structure of the reference bulk for GaP and GaSb supercells, with bandgaps of 2.23 and 1.75 eV respectively. For a clear comparison, the Valence Band Maximum (VBM) and the Conduction Band Minimum (CBM) of the bulk-supercells were marked in Fig. S25a and Fig.

S25b, shown as the horizontal white dotted lines. All the structures show band folding effect due to the construction of the supercell. As compared to the bulk, the APB structures do not affect much the nature of the CBM while they introduce two localized states at the top of the VB, shifting the VBM upward (by roughly 0.5 eV for GaP and 0.3 eV for GaSb) and thus reducing the bandgap, in good agreement with data reported in ref. [1,6].

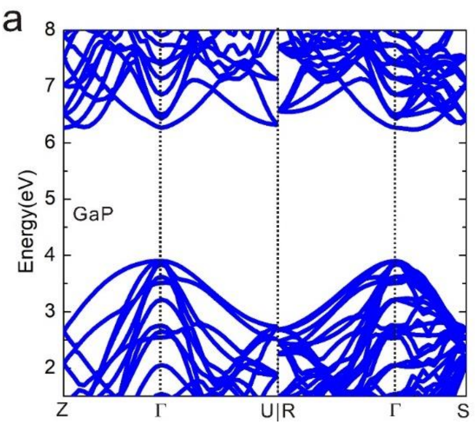

Figure S24: Band structures of zinc-blende bulk GaP (a) and GaSb (b) corresponding to Fig. S9a.

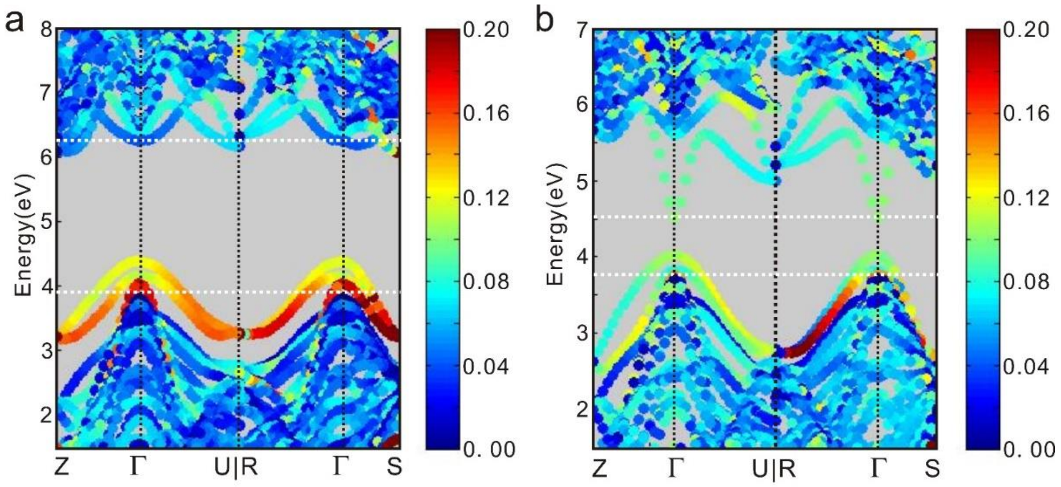

Figure S25: Band structures of the stoichiometric APB structures of GaP (a) and GaSb (b) corresponding to Fig. S9b.

Analysis about higher photo-electrochemical activity of III-V samples grown on n (p)-doped Si substrate in the anodic (cathodic) region

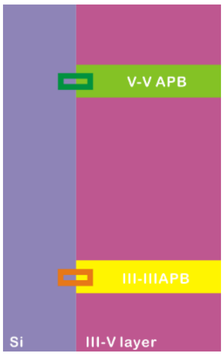

Figure S26: Schematic diagram of a bi-domain III-V/Si sample with III-III and V-V APBs. The interfaces of Si with V-V APB and Si with III-III APB are marked by the green and orange boxes, respectively.

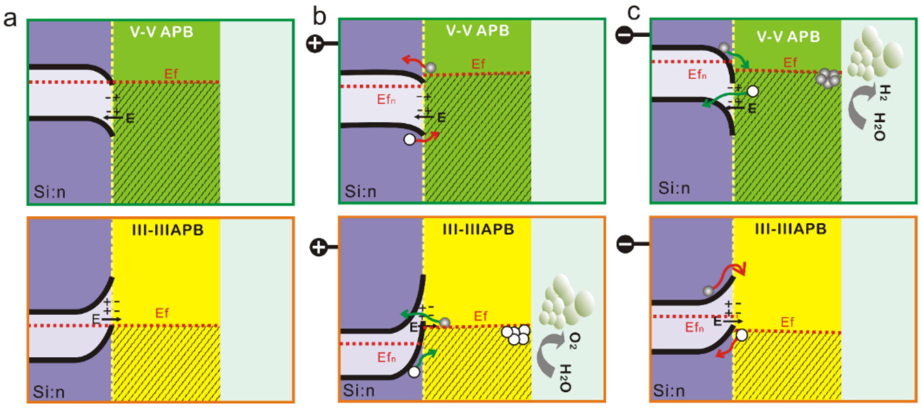

Figure S27: Local band bendings between Si and III-III or V-V APBs of the GaP/Si:n samples in three conditions: without bias (a), under positive bias (b) and under negative bias (c), proposed to explain the higher anodic photocurrent and lower cathodic photocurrent as shown in the voltammetry curve (Fig. 2d). The small subgraphs with green and orange enclosed boxes correspond to the interfaces of Si with V-V APB and Si with III-III APB, respectively.

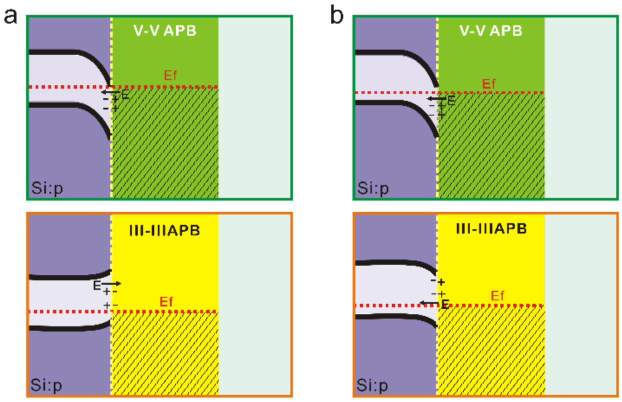

Figure S28: Local band bendings between Si and III-III or V-V APBs of the GaPSb/Si:p sample, proposed to explain the higher cathodic photocurrent and lower anodic photocurrent as shown in the voltammetry curve (Fig. S7). The Fermi level of Si is either between the Fermi levels of III-III and V-V APBs and closer to the III-III's one (a) or even below both III-III and V-V Fermi energies (b). The small subgraphs with green and orange enclosed boxes correspond to the interfaces of Si with V-V APB and Si with III-III APB, respectively.

Based on the voltammetry curves, the III-V samples on Si:n substrate (GaP/Si:n, GaPSb/Si:n and GaPAs/Si:n) show higher anodic photocurrent (Fig. 2d-f) and the III-V sample on p-doped Si substrate (Si:p) (GaPSb/Si:p) exhibits higher cathodic photocurrent (Fig. S7). In order to clarify this, the local band bendings between n-doped and p-doped Si substrates with III-V layer need to be analyzed and the schematic drawings are displayed in Fig. S27 for GaP/Si:n and Fig. S28 for the GaPSb/Si:p, where the small subgraphs with green and orange enclosed boxes correspond to the interfaces of Si with III-III APB and Si with V-V APB, respectively (Fig. S26). Fig. S27a shows the band bendings between Si:n and III-III APB or V-V APB without applied bias. From Fermi levels lineups (Fig. 4), we know that the Fermi energy level of n-doped Si locates between the V-V and III-III Fermi levels of the GaP/Si:n sample, which causes the bands of Si to bend downwards and upwards to V-V and III-III APB structures, respectively. It must be noticed that, the Fermi energy level of n-doped Si being closer to the one of V-V APB, the band bending between Si and V-V APB is weaker than the band bending between Si and the III-III APB. When a positive voltage is applied to

Si, the strong band bending between Si and III-III APB facilitates the carrier transport, as illustrated by the green arrows in Fig. S27b, and the weak band bending between Si and III-III APB becomes a small barrier for carriers transfer (illustrated by the red arrows in Fig. S27b), which can disappear easily with the increase of the voltage, leading to relatively large anodic photocurrent. At the opposite, when negative voltage is applied to Si, the relatively strong band bending between Si and III-III APB becomes the barrier for carrier transfer (illustrated by the red arrows in Fig. S27c), which causes relatively small cathodic photocurrent. For the III-V sample on p-doped Si substrate (GaPSb/Si:p sample), the situation is reversed. The Fermi energy level of p-doped Si is closer to the one of III-III APB (Fig. S28a) or even below the Fermi level of III-III APB (Fig. S28b) based on the Fermi energy calculation results (Fig. 4m,n,o). This situation results in a stronger band bending between Si and V-V APB, which acts as a large barrier for carrier transport under positive voltage on Si, giving rise to relatively low anodic photocurrent. When the Fermi level of Si is below both III-III and V-V Fermi levels (Fig. S28b), both the band bendings between Si and III-III or Si and V-V will facilitate cathodic photocurrent and impede anodic photocurrent, leading to a larger difference between cathodic and anodic photocurrents. Besides, in this case, the PEC response of the Si substrate can be activated.

Analysis about the light absorption contribution of the underlying Si substrate to the overall PEC response

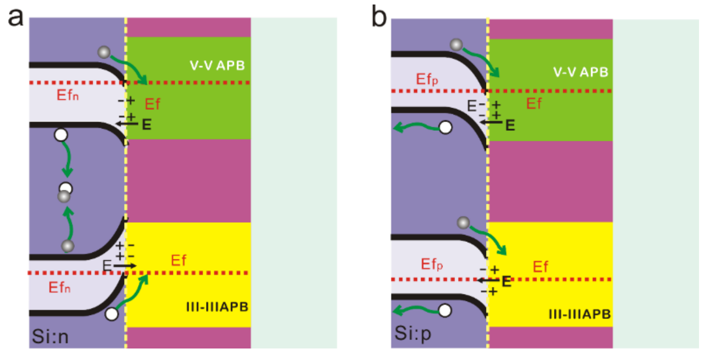

Figure S29: Scheme explaining the light-absorption contribution of the different Si substrates to the PEC response for the III-V sample with n-doped Si substrate (i.e. the Si Fermi level lies between the Fermi levels of III-III and V-V APB structures) (a) and with a p-doped Si substrate (where the Si Fermi level lies below both the III-III and V-V Fermi levels) (b).

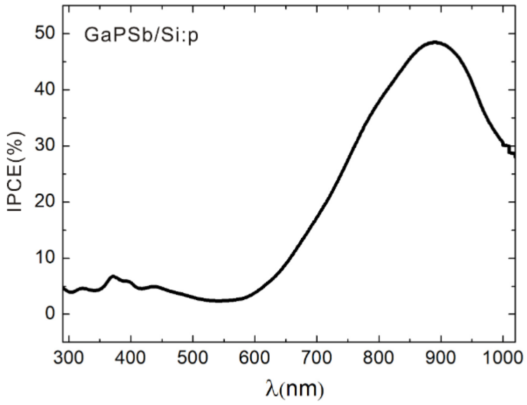

Figure S30: IPCE spectrum recorded at -0.74 V (cathodic region) for the bi-domain GaPSb/Si:p sample, which shows a large signal between 600 and 1000nm (corresponding to Si absorption).

Based on the local band bendings of Si with III-III and V-V APBs at the interfaces, we can also make a discussion explaining why the absorption of the n-doped Si substrate doesn't contribute to the PEC response for the GaP/Si:n sample (any significant IPCE signal was not observed below 750 nm in Fig. 2c). As discussed above, if the Fermi level of n-doped Si lies between the Fermi levels of III-III and V-V APB structures, the bands of Si will bend downward and upward with V-V and III-III APB structures, respectively. In this situation, under an incident light in the 750-1100nm wavelength range, only the Si substrate absorbs the

light and generates electron/hole pairs. Due to the inverted band bendings between Si and V-V or III-III APB, the photo-generated electrons close to the Si/V-V interface will easily flow to the GaP layer through a V-V APB leaving the holes in Si. The photo-generated holes close to the Si/III-III interface will easily access to the GaP layer through a III-III APB structure, leaving the electrons in Si. This will lead to electrons/holes recombinations, as shown in Fig. S29a. Therefore, the light absorption of the n-doped Si (for the GaP/Si:n sample) doesn’t give an obvious PEC response.

On the other hand, if the Fermi energy of Si is below or above the Fermi levels of both III-III and V-V APB structures, then the same band bendings between Si and a V-V APB or Si and a III-III APB will occur. For example, this configuration is achieved for GaSb or GaSb-based alloys grown on Si:p substrates (the Fermi level can be lower than both III-III and V-V APB structures). In this situation, the band of Si will bend downward with both V-V and III-III APB structures (Fig. S29b). The photo-generated electrons in Si will flow to III-V layer and the holes will stay in Si, leading to charge separation and contribute to the IPCE response, as shown in Fig. S29b. This was evidenced by the IPCE spectrum of the GaPSb/Si:p sample, which shows a large IPCE signal in the 600-1000 nm wavelength range (mainly corresponding to Si absorption), as shown in Fig. S30.

The local band bendings between Si and the III-III or V-V APB at the III-V/Si interfaces provide degrees of improvements to optimize charge carrier transfers, or even by making both Si substrate and bi-domain III-V layer photoactive during PEC operation with a careful optimization of Fermi levels lineups through both III-V alloys composition or Si substrate doping.

General picture of bi-domain III-V/Si material with ambipolar transport and PEC properties

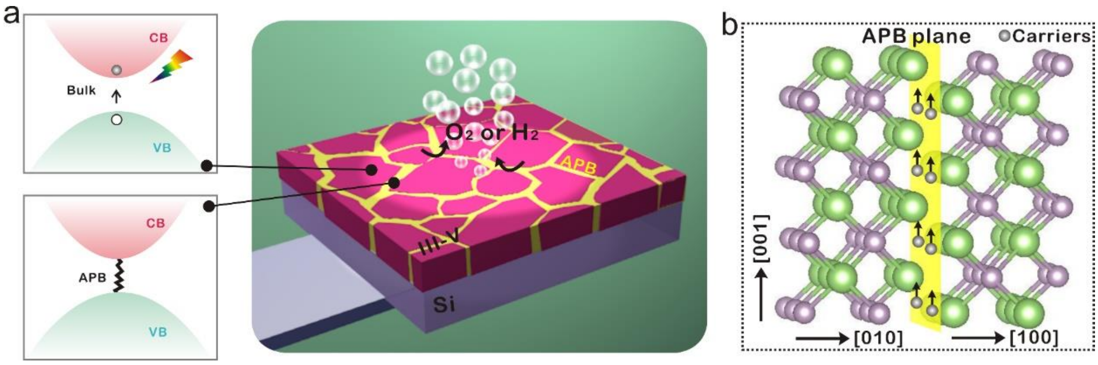

Figure S31: a. General illustration of the bi-domain III-V/Si material composed of bulk photo-active semiconductors with 2D APB semi metallic inclusions, realizing ambipolar PEC water splitting. b. Schematic plot of carriers' transport along the 2D APB planes at the atomic scale.

Fig. S31 shows the general picture of bi-domain III-V/Si material with hybrid 2D semimetal/semiconductor properties enabling photo-generation through optical absorption and good vertical transport, with ambipolar PEC properties.

References

[1] L. Chen, O. Skibitzki, L. Pedesseau, A. Létoublon, J. Stervinou, R. Bernard, C. Levallois, R. Piron, M. Perrin, M. A. Schubert, A. Moréac, O. Durand, T. Schroeder, N. Bertru, J. Even, Y. Léger, C. Cornet, *ACS Nano* **2020**, *14*, 13127.

[2] Y. Ping Wang, A. Létoublon, T. Nguyen Thanh, M. Bahri, L. Largeau, G. Patriarche, C. Cornet, N. Bertru, A. Le Corre, O. Durand, *J. Appl. Cryst.* **2015**, *48*, 702.

[3] R. Saleem-Urothodi, J. Le Pouliquen, T. Rohel, R. Bernard, C. Pareige, A. Lorenzo-Ruiz, A. Beck, A. Létoublon, O. De Sagazan, C. Cornet, Y. Dumeige, Y. Léger, *Opt. Lett.* **2020**, *45*, 4646.

[4] B. Yan, C. Felser, *Annu. Rev. Condens. Matter Phys.* **2017**, *8*, 337.

[5] S.-Y. Yang, H. Yang, E. Derunova, S. S. P. Parkin, B. Yan, M. N. Ali, *Adv. Phys. X* **2018**, *3*, 1414631.

[6] E. Tea, J. Vidal, L. Pedesseau, C. Cornet, J.-M. Jancu, J. Even, S. Laribi, J.-F. Guillemoles, O. Durand, *J. Appl. Phys.* **2014**, *115*, 063502.
